# A Spike-Accum bioconjugate protein vaccine confers potent SARS-CoV-2-specific immunity

**DOI:** 10.1016/j.isci.2025.113314

**Published:** 2025-08-07

**Authors:** Jean Pierre Bikorimana, Nathanael A. Caveney, Nehme EL-Hachem, Gabrielle A. Mandl, John A. Capobianco, Daniela Stanga, Jamilah Abusarah, Mark A. Hancock, Roudy Farah, Marina P. Gonçalves, Darryl Falzarano, Mingmin Liao, Glenn Hamonic, Qiang Liu, Simon Beaudoin, Sebastien Talbot, Moutih Rafei

**Affiliations:** 1 Department of Microbiology, Infectious Diseases, and Immunology, Université de Montréal, Montreal, QC H3T 1J4, Canada; 2 Centre for Blood Research, University of British Columbia, Vancouver, BC V6T 1Z3, Canada; 3 Department of Pharmacology and Toxicology, University of Toronto, Toronto, ON M5S 1A8, Canada; 4 Sainte-Justine Research Centre, Montreal, QC H3T 1C5, Canada; 5 Department of Chemistry and Biochemistry and Centre for NanoScience Research, Concordia University, Montreal, QC H3T 1J4, Canada; 6 Research and Development Branch, Defence Therapeutics Inc., Montreal, QC H4S 1Z9, Canada; 7 Department of Pharmacology and Physiology, Université de Montréal, Montreal, QC H3T 1J4, Canada; 8 SPR-MS Facility, Department of Pharmacology and Therapeutics, McGill University, Montreal, QC H3G 1Y6, Canada; 9 Vaccine and Infectious Disease Organization, University of Saskatchewan, Saskatoon, SK S7N 5E3, Canada; 10 Department of Biomedical & Molecular Sciences, Queen’s University, Kingston, ON K7L 3J8, Canada; 11 Molecular Biology Program, Université de Montréal, Montreal, QC H3T 1J4, Canada

**Keywords:** Natural sciences, Biological sciences, Immunology, Immune response

## Abstract

Despite the recent control of COVID-19, the devastating effects caused by the 3-year pandemic highlight the importance of developing effective vaccines to rapidly address future outbreaks. The present study describes a novel Spike (Sp) protein-based vaccine formulation using the Accum platform. Although Sp-Accum bioconjugation does not alter the overall protein structure, it triggers a substantial antibody titer: i) exhibiting higher specificity toward the S1 domain of Sp, ii) neutralizing Sp-ACE2 interactions, and iii) cross-reacting with various Sp variants. Besides validating the vaccine immunogenicity in rabbits, its administration in a “gold-standard” SARS-CoV-2 hamster model was shown to be safe while accelerating viral clearance without eliciting signs of pathological inflammation in the lungs of infected animals. Altogether, this proof-of-concept study not only demonstrates once again the versatility of the Accum technology in vaccine engineering, but it provides an enabling technology for the rapid development of value-added, protein-based vaccines for future pandemics.

## Highlights

• Accum bioconjugation enhances the immunogenicity of the Spike protein
• Generated antibodies neutralize Spike-ACE2 interactions and cross-react with COVID variants
• The Spike-Accum vaccine accelerates viral clearance from infected hamsters
• Hamsters vaccinated with Spike-Accum show no major signs of lung inflammation

## Graphical abstract

**Figure:**
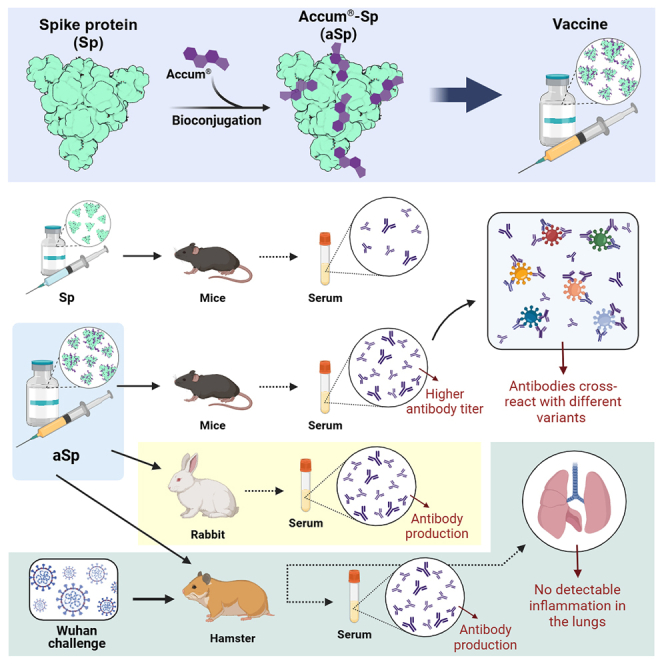

## Introduction

With more than 700 million infections worldwide, the 2020–2023 pandemic caused by the Severe Acute Respiratory Syndrome Coronavirus 2 (SARS-CoV-2) has underscored the importance of developing rapid, safe, and effective vaccines.^1^ SARS-CoV-2 is an RNA virus enveloped by crown-like proteins, known as Spike (Sp) proteins (⁓25 proteins/capsid), a feature common to all coronaviruses.^2^^,^^3^ The Sp protein consists of an S1 domain comprising the receptor binding domain (RBD) and an S2 domain, both playing major roles in modulating virus-host interactions and entry into host cells via the angiotensin converting enzyme 2 (ACE2) receptor.^4^^,^^5^^,^^6^ Since Sp is crucial for the pathogenicity of the virus, it has become a highly attractive target for vaccine development.^7^^,^^8^^,^^9^ For instance, serological analyses conducted on infected patients revealed that up to 90% of neutralizing antibodies target the S1 RBD-ACE2 binding interaction.^10^^,^^11^ These observations, combined with the devastating health, social, and economic impacts of the virus, led to the urgent fast track and approval of COVID-19 mRNA vaccines.^12^^,^^13^^,^^14^ These vaccines provide an mRNA template encoding for the Sp protein to be produced by host cells, which is then captured and presented by immune cells to generate an anti-Sp targeted immune response.^15^^,^^16^^,^^17^^,^^18^

In general, mRNA-based vaccines are particularly attractive for eliciting rapid immunity while requiring relatively fast manufacturing and production steps once the target sequence is known.^18^^,^^19^^,^^20^^,^^21^ However, these vaccines are still associated with important hurdles, including weak to moderate humoral responses due to inconsistent and nonspecific uptake of mRNA molecules by muscle cells, and molecular barriers such as endosomal entrapment hindering mRNA access to the cytosol.^21^^,^^22^ Moreover, additional vaccine boosters are needed to address the waning of cellular and humoral immune responses with time.^20^^,^^23^^,^^24^^,^^25^ Logistically, the cryogenic temperatures required to preserve mRNA vaccines presented a significant obstacle during mass COVID-19 vaccination campaigns.^20^^,^^26^

Protein-based SARS-CoV-2 vaccines could bypass some of the limitations noted above, thereby representing an attractive vaccine strategy against COVID-19. Notably, several COVID-19 protein subunit vaccine candidates were recently approved.^27^^,^^28^ Despite their later adoption, these vaccines provided long-term protection while maintaining a favorable safety profile.^12^^,^^27^^,^^29^ To capitalize on these advantages, novel approaches to further improve the generated protective immune response have included the use of different protein expression systems, developing and/or optimizing adjuvants, and protein nano-encapsulation among other means to further improve their delivery to target cells.^30^^,^^31^^,^^32^^,^^33^ One of the strategies adapted to enhance antigen delivery consists of bioconjugating Sp to Accum, a molecule composed of a cholic acid fused to a nuclear localization signal designed to facilitate protein escape into the cytosol to, in turn, enhance target protein bioaccumulation in target cells.^34^^,^^35^^,^^36^ In fact, multiple groups have reported the therapeutic potency of this strategy in the context of antibody-drug conjuagtes.^34^^,^^35^^,^^36^^,^^37^^,^^38^ Moreover, we have previously exploited the parental Accum molecule and one derivative for the design of next generation cell cancer vaccine candidates.^39^^,^^40^ By ensuring earlier release from endosomes, target antigens are prevented from excessive and/or non-specific degradation thus resulting in efficient processing by the proteasomal complex.^34^^,^^36^^,^^37^^,^^38^ Consequently, the improved concentration and quality of derived peptides available for immune priming and activation have been demonstrated with a dendritic cell-based vaccination pulsed with a defined or protein lysate conjugated to Accum or using the human papilloma virus Accum-E7 bioconjugate oncoprotein.^39^^,^^40^

Based on the previous findings reported in the literature noted above, we elected to further explore the advantages of bioconjugating Accum onto the Sp protein (aSp). In this context, we found that aSp retained its overall protein structure yet induced powerful humoral responses capable of neutralizing the ACE2-Sp interaction and thus viral infectivity. While antibodies induced by this vaccine cross-reacted with various COVID-19 RBD variants, the potency of aSp translated into accelerated infection clearance along with a strong host resistance to lung inflammation as shown in a SARS-CoV-2 hamster model. Overall, our present study advances the development of Accum-bioconjugated, protein-based vaccines to rapidly address future outbreaks.

## Results

### Accum bioconjugation does not alter the overall structure of trimeric spike

**Figure 1:**
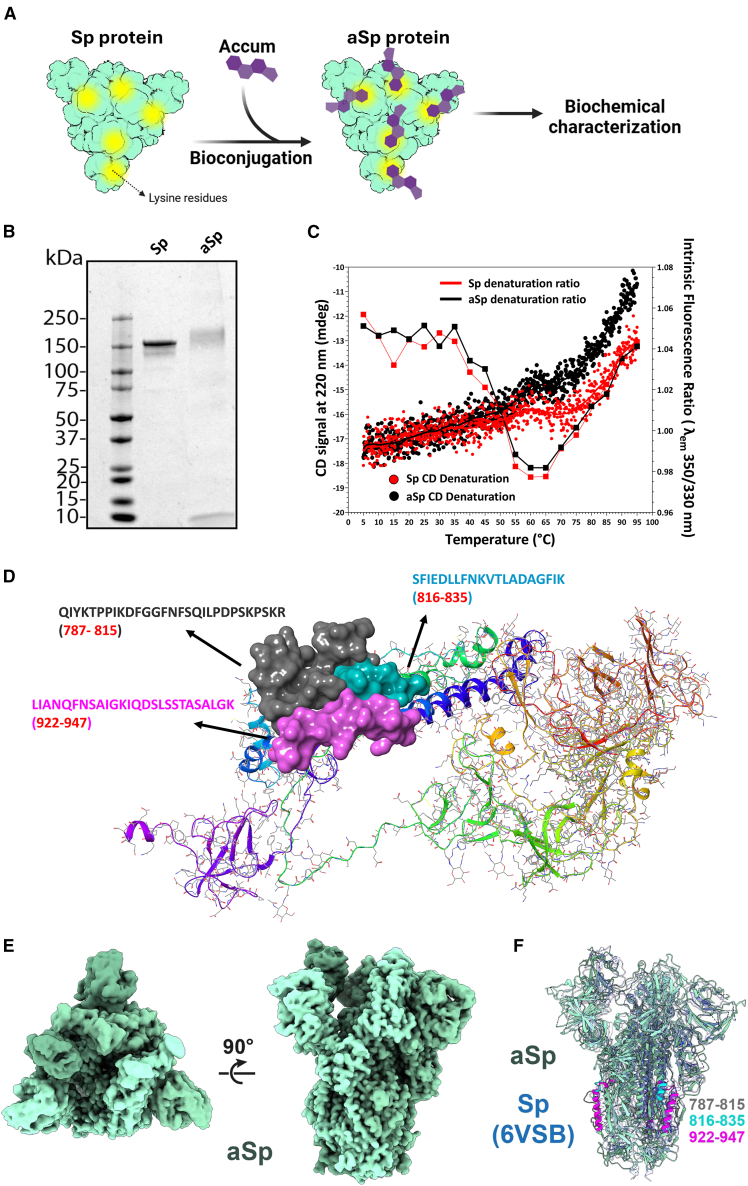
Biochemical characterization of the engineered aSp vaccine (A) Schematic of lysine-specific bioconjugation of Accum to Spike protein to generate candidate aSp vaccine. (B) Representative 4–12% SDS-PAGE under reducing conditions with Coomassie staining to compare the molecular weight of unmodified Sp versus modified aSp. (C) Representative temperature-dependent CD spectroscopy versus tryptophan fluorescence of Sp (black) and aSp (red) proteins. (D) Predicted model of Sp protein with three candidate derivation sites that were detected by mass spectrometry. (E) Cryogenic-electron microscopy (cryoEM) reconstruction: aSp protein is colored in green versus various shades thereof for each monomer within the Sp trimer. (F) Structural comparison of the model built and refined against the aSp reconstruction overlaid on the structure of unmodified Spike (PDB 6VSB).^41^ The aSp is colored as in E, with regions additionally colored on each of the three monomers, as determined in panel D. The unmodified Sp protein is partially transparent and colored in various shades of blue. Also see Figures S1–S3, and Table S1.

The parent Accum molecule is a small lipopeptide composed of a cholic acid linked to the SV40 nuclear localization signal.^36^ This molecule can be conjugated onto any protein via its exposed lysine residues (Figure 1A). To confirm that our Sp bioconjugation was successfully completed using a sulfo-SMCC linker, the unmodified (Sp) and modified (aSp) proteins were compared by SDS-PAGE which revealed that aSp migrated at a slightly higher molecular mass, as expected (Figure 1B).

Temperature-dependent circular dichroism (CD) spectroscopy enables the facile evaluation of protein stability, particularly by analysis of signals in the far-UV region (190–250 nm).^42^ The CD signal at 220 nm corresponds to the n→π∗ electronic transition of peptide bonds and is highly sensitive to changes in the alpha helices and beta sheets within the protein, allowing for monitoring of protein secondary structure in response to changes in the environment, such as temperature or pH, for example. Our CD analyses encompassed a wide temperature range (5°C–95°C) to evaluate the thermal stability of aSp versus the unmodified Sp protein across temperatures relevant to physiology, transport, and storage. As shown in Figure 1C (connected curves), substantial protein denaturation began around 40°C for both proteins, indicating that the Accum conjugation process did not negatively impact thermal stability. Since the Sp protein can exhibit different conformations, which are known to be susceptible to pH changes (especially in acidic-neutral regime), similar CD analyses were performed to assess the thermal stability of both proteins as a function of pH and chemical denaturation.^43^^,^^44^ As shown in Figure S1A, Sp exhibits minimal conformational changes between pH 3 to 10, with similar denaturation observed around 60°C, confirming the stability of the Sp protein in both acidic and alkaline conditions. Interestingly, aSp behaved differently, with increased stability at higher pH (7, 9, and 10) as evidenced by a less-pronounced decrease in CD signal at 55°C (Figure S1B). Furthermore, an independent assessment of protein thermal stability was conducted using ratiometric analysis of intrinsic (tryptophan) fluorescence at 350 and 330 nm as a function of temperature. Higher ratios are indicative of a predominately hydrophobic environment, suggesting that the tryptophan residues within a protein are better shielded from the external environment across all temperatures, regardless of protein denaturation. As expected, the cholic acid moiety of Accum appears to impart an increase in hydrophobicity to aSp (compared to unmodified Sp), as evidenced by the higher 350/330 nm ratio across most of the temperatures studied (Figure 1C - cloud of dots).

To further characterize the modified aSp protein, we next sought to identify the site(s) of Accum bioconjugation onto the Sp protein. Using liquid chromatography-tandem mass spectrometry, five peptide sequences were identified as sites of modification (Figure S2; Table S1), yielding an average drug-antibody ratio of 3.3. Of these five sequences, three distinct amino acid regions (787–815, 816–835, and 922–947) were observed as being the regions preferentially targeted for modification, likely due to preferential steric exposition favoring their reactivity (Figure 1D). To confirm that Accum bioconjugation to these sites did not alter the overall protein structure, cryogenic-electron microscopy (cryoEM) was used. A reconstruction was generated that contains clear density for most of the proteinaceous components of the Sp ectodomains (Figures 1E and 1F). As observed in many unmodified structures,^41^^,^^45^^,^^46^ native Sp was largely seen to exist in an open conformation, with one RBD extending upward away from the central trimer. In comparison to the unmodified Sp structure (PDB 6VSB),^47^ we calculate an RMSD of ∼1.4 Å over ∼950 core atom pairs, or 2.9–3.5 Å across all atom pairs within a single chain. Further assessment of native and Spike-Accum using three different commercially available monoclonal antibodies (mAb) (clone B3476M; clone B3478M; and clone 1C3H9) via ELISA confirms that the conjugation does not affect the binding of mAb (Figure S3). In summary, the biochemical analyses above clearly demonstrate that Accum bioconjugation does not significantly alter the overall Sp protein structure, yet confers modest resistance to temperature-mediated denaturation.

### The bioconjugated aSp vaccine elicits a humoral response superior to unmodified spike

**Figure 2:**
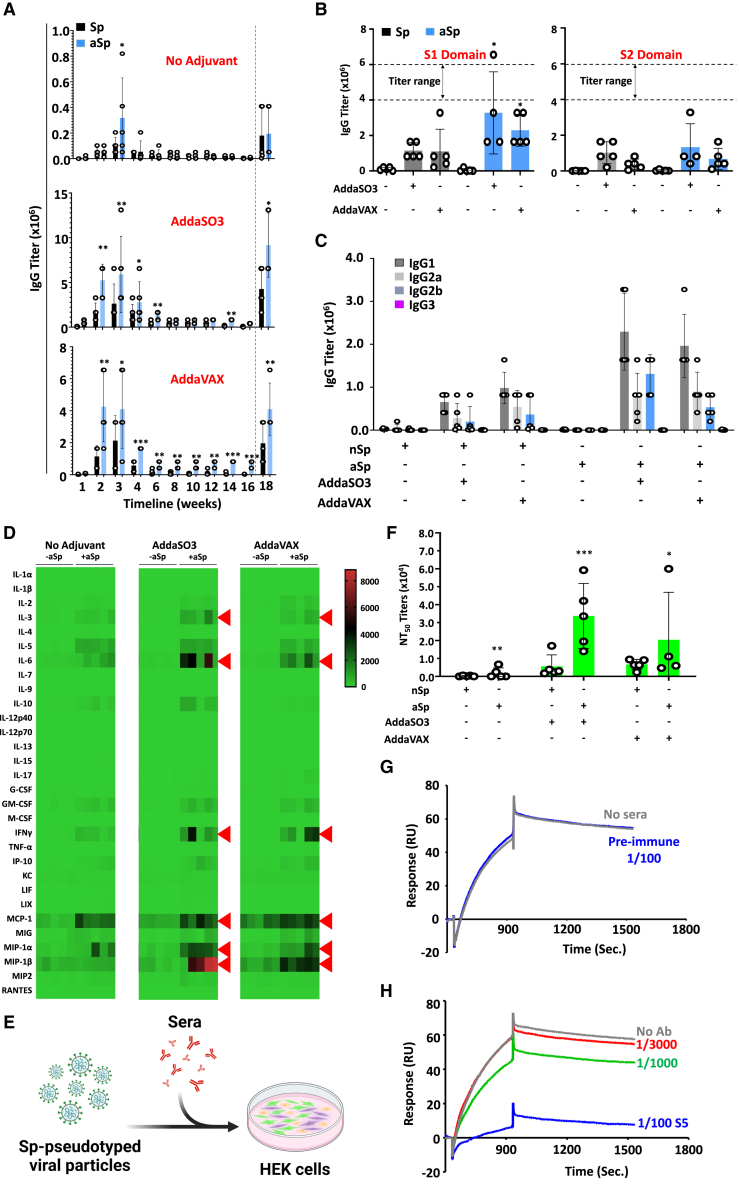
Comparing the immunogenicity of Sp and aSp in immunocompetent mice, as assessed using different adjuvants (A) ELISA assessment of IgG titers over 18 weeks total post-immunization. The dotted red line represents the time point at which a recall vaccination (third dosing) was conducted in the absence of adjuvant. (B) ELISA assessment of IgG binding to the S1 versus S2 domain of the Sp protein. The sera analyzed in this experiment were derived from samples collected at week 3. (C) ELISA assessment of the different isotypes contained in the sera derived from animals receiving the Sp or aSp vaccine. The samples analyzed were those collected at week 3. (D) Luminex analysis conducted on *in vitro* re-stimulated splenocytes immunized using the same schema depicted in panel A. Red triangles refer to cytokines/chemokines with depicted differences. (E) A representative schema of the *in vitro* infectivity assay conducted on HEK cells using Sp-pseudotyped viral particles in the presence of neutralizing antibodies derived from the experiment shown in panel B. (F) The NT_50_ results of the infectivity assay conducted in the presence of the neutralizing antibodies. (G) Representative SPR sensorgram in which capture of pre-immune sera (1/100 dilution as negative control) failed to block ACE2 binding to Sp-immobilized sensors. (H) SPR in which 1/100 or 1/1000 dilutions of week 3 neutralizing antibody (nAb)-containing sera #5 inhibited ACE2-Sp binding; the effect was lost when the nAb sera was diluted 1/3000. For experiments in panels A-F: n = 5–6 per group. Data are represented as mean ± SD with ∗*p* < 0.05, ∗∗*p* < 0.01 and ∗∗∗*p* < 0.001. Also see Figures S4–S7.

Since the objective of a given vaccine is to elicit a humoral response capable of blocking viral infectivity, we first conducted a full spectrum analysis of antibody titers triggered in response to unmodified Sp versus aSp immunization. Accordingly, pre-immune sera were collected from immunocompetent C57BL/6 mice prior to prime (day 0) and boost (day 14) immunizations. Blood samples were then collected on a weekly basis for the first 4 consecutive weeks followed by sera collection every 2 weeks for a total period of 18 weeks. In the same experiment, we also compared the use of two different adjuvants: AddaSO3 (an oil-in-water nano-emulsion adjuvant) and AddaVAX (a squalene-based oil-in-water nano-emulsion); both capable of eliciting mixed cell-mediated and humoral immune responses. Compared to animals vaccinated in the absence of adjuvants, higher titers were observed mostly within the first 4 weeks post-boosting in the aSp group, irrespective of the adjuvant used (Figure 2A). Given that titer levels obtained after the third and final boost at week 17 (red dotted line) were similar in magnitude to titers obtained at week 3 or 4, this suggests that the use of a prime/boost (P + B) immunization scheme is sufficient to trigger a peak response against Sp (Figure 2A). Interestingly, most of the generated antibodies showed higher binding selectivity toward the S1 domain of the unmodified Sp protein (especially those derived from aSp-immunized mice - Figure 2B). Isotype analysis revealed the predominant presence of IgG1 along with IgG1a and IgG2b with barely any detectable IgG3, suggesting a predominant T-helper 1 response (Figure 2C). This is consistent with the production of interferon-gamma (IFN-γ) from *in vitro* re-stimulated splenocytes in the absence of major T-helper 2 cytokines such as interleukin (IL)-4 and IL-5 (Figure 2D). Since IgG1 antibodies are known to exhibit powerful neutralization and opsonization effects, their predominant production in this context is ideal due to their high selectivity toward the S1 domain of Sp (containing the RBD region).

To further highlight the potency of the generated antibodies, we next tested their neutralizing capacity using an *in vitro* infectivity assay on HEK cells cultured with Sp-pseudotyped viral particles (Figure 2E). Sera derived from aSp/AddaSO3-immunized animals resulted in the highest 50% neutralizing titer (NT_50_) followed by the aSp/AddaVAX-vaccinated group (Figure 2F). Besides this cell-based assay, we next developed a robust surface plasmon resonance (SPR) assay to study the neutralizing efficacy of the generated antibodies on the ACE2-Sp binding interaction. While titrating the Sp protein against ACE2-immobilized sensors was specific and dose-dependent, we found it was difficult to regenerate the reference and ACE2-immobilized surfaces in this configuration (data not shown). Alternatively, the reversed “analyte-ligand” orientation (i.e., injecting ACE2 over Sp-immobilized sensors) yielded superior titration/regeneration conditions (Figure S4) that correlated well with ACE2-Sp binding constants previously reported in the literature (i.e., high-affinity interaction with slow dissociation rate and low nM equilibrium dissociation (*K*_D_) constant overall).^46^ Before testing if the generated antibodies could block binding between Sp and ACE2, a preliminary “clean screen” assay was performed by flowing the sera samples over reference-only surfaces (i.e., activated/blocked dextran without immobilized protein). Compared to the purified ACE2 injections (100 nM), which exhibited minor non-specific binding (Figure S4A, <5 RU dashed arrow), the minimally diluted sera samples #1–5 (100-fold) exhibited low non-specific binding despite their heightened sample complexity (<60 RU, solid arrow). The subsequent injection series over in-tandem ACE2-and Sp-immobilized surfaces showed that the sera-derived antibodies were captured to Sp surfaces only, thus blocking subsequent ACE2 binding (Figure S4B). The developments above led to our optimized SPR assay in which back-to-back capture (e.g., sera at fixed 1/200 dilution) and binding (100 nM ACE2) phases allowed us to compare different neutralizing antibody sera #1–5 for their ability to block the ACE2-Sp binding interaction (Figure S5). Notably, capturing pre-immune sera (modest 1/100 dilution) failed to block the ACE2-Sp binding (Figure 2G). On the other hand, serially diluted antibody-containing sera showed that the 1/100 and 1/1000 dilutions inhibited ACE2-Sp binding, but their neutralization effects were absent when diluted to 1/3000 (Figure 2H; Figure S6). In summary, the results above indicate that aSp immunization i) elicits a stronger immune response than unmodified Sp, ii) favors the production of IgG1 antibodies that predominantly bind to the S1 domain of Sp, and iii) is capable of inhibiting the viral infection of mammalian cells *in vitro*.

### Antibodies induced by aSp cross-react with other SARS-CoV-2 variants

**Figure 3:**
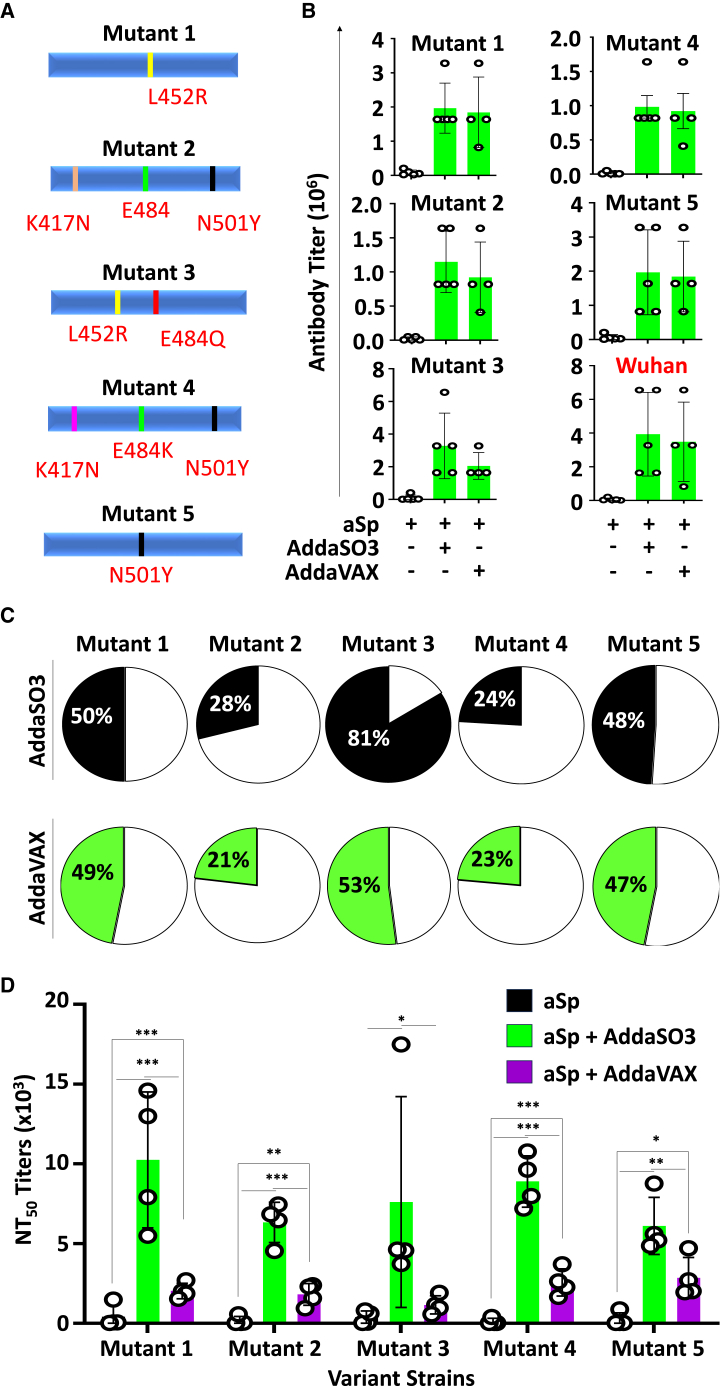
The antibodies generated using the aSp vaccine cross-react with different Sp RBD variants (A) Schematic representing the different Sp RBD variants tested in our study. (B) Representative ELISAs assessing the cross-reactivity of the sera (week 3) derived from the immunization study shown in panel 2B. (C) Pie charts summarizing the neutralizing capacity of the antibodies tested in panel B. (D) NT_50_ results for the infectivity assay conducted using different Sp variant-pseudotyped viral particles in the presence of neutralizing antibodies. For panels B and D, *n* = 5 and 4, respectively. Data are represented as mean ± SD with ∗*p* < 0.05, ∗∗*p* < 0.01, and ∗∗∗*p* < 0.001.

Due to the fast evolution of the COVID-19 virus and the appearance of different mutations worldwide, we then tested the reactivity of our aSp-induced sera toward five different SARS-CoV-2 RBD variants (Figure 3A). Compared to the original non-mutated Wuhan RBD, collected sera reacted with all five tested proteins, irrespective of the used adjuvant, albeit at different efficacy (Figure 3B). More specifically, we observed a superior binding of aSp-derived sera to RBD with L452R/E484Q mutations (mutant 3) followed by L452R (mutant 1), and then N501Y (mutant 5; Figure 3C). Weaker but detectable binding could also be observed with RBD containing the K417N/E484/N501Y (mutant 2) and K417N/E484K/N501Y (mutant 4; Figure 3C). To further validate these observations, we conducted another *in vitro* infectivity assay using HEK cells exposed to Sp-pseudotyped viral particles containing the same mutations. Interestingly, all tested sera derived from mice immunized with aSp/AdsaSO3 were not only superior in inhibiting HEK cell infection compared to the AddaVAX group, but the rate of inhibition was similar among all groups (Figure 3D). The latter observation suggests that the differences in the cross-reactivity observed with the different strains does not necessarily correlate with better neutralizing efficacy.

### The aSp vaccine is immunogenic in non-rodent animals

**Figure 4:**
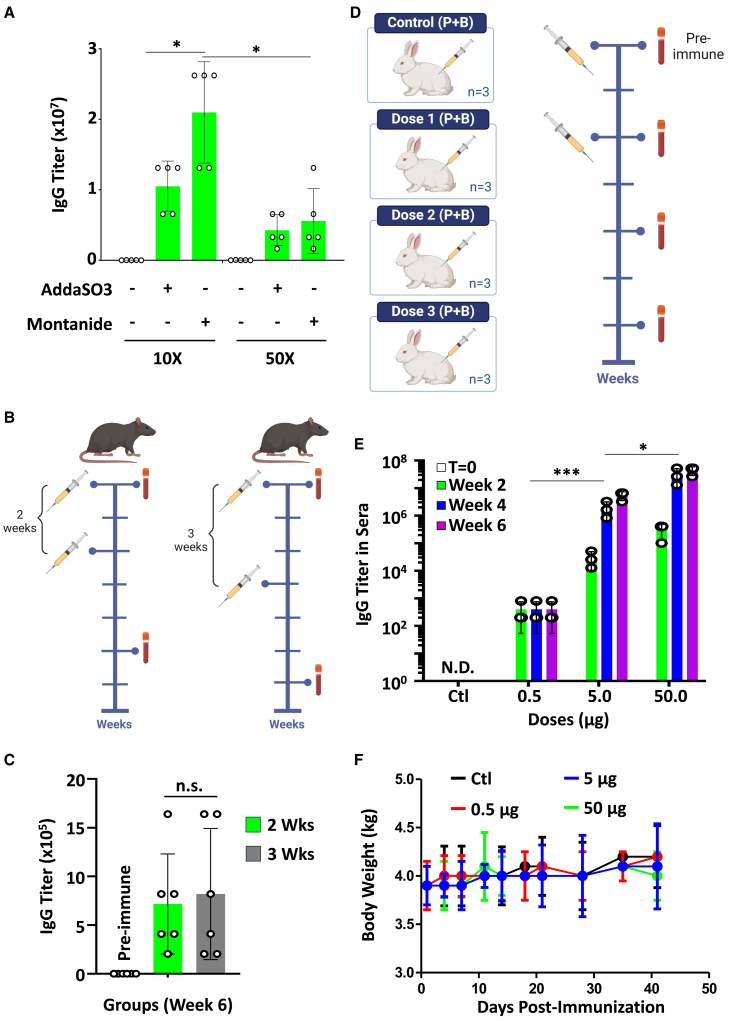
Comparative immunogenicity of the aSp vaccine in rodents and rabbits (A) Evaluating the humoral response of two different aSp vaccines (10X versus 50X) using two different adjuvants. (B) Schematic depicting the different dosing schemes tested in immunocompetent mice. (C) IgG titers quantified by ELISA using sera isolated three weeks following the boost in both immunization schedules. (D) Schematic depicting the different (P + B) dosing schemes tested in rabbits (non-GLP study). (E) IgG titers quantified by ELISA using sera isolated at different time points from all immunized animals. (F) Assessment of body weights of all immunized rabbits over a period of 42 days. For panels A, *n* = 5/group. For panel C, *n* = 6/group and for panels E-F, *n* = 3/group. For this figure, data are represented as mean ± SD with ∗∗*p* < 0.01 and ∗∗∗*p* < 0.001.

Our preliminary immunogenicity tests of aSp were assessed in immunocompetent mice using two pre-clinical adjuvants. To ensure the translational aspect of the vaccine, we next conducted an immunization study comparing AddaSO3 to the FDA-approved GMP grade Montanide ISA 720 VG adjuvant.^11^^,^^48^ In our study, two variables were investigated: i) engineering aSp vaccines using a 10- versus 50-fold excess Accum to identify the optimal bioconjugation condition, and ii) comparing the magnitude of the humoral response to these protein-based vaccines using AddaSO3 versus Montanide ISA 720 VG. When vaccinated using the same regimen previously used in immunocompetent C57BL/6 mice, two observations were made. First, the use of the Montanide ISA 720 VG adjuvant was superior to AddaSO3 in terms of antibody titers (Figure 4A). Second, the immunogenicity of the 10X Accum to protein ratio was superior to the 50X excess condition (Figure 4A). Considering these observations, we next sought to assess whether the prime/boost (P + B) vaccination regimen used so far is optimal by comparing the antibody titer when the boost is administered 2 versus 3 weeks post-vaccine priming (Figure 4B). Since no major differences were observed in the measured antibody titers (Figure 4C), we next conducted a non-GLP immunization study in rabbits using the two-week interval. Briefly, four groups of rabbits were used: i) control P + B using Montanide ISA 720 VG adjuvant alone, ii) P + B using 0.5 μg of the vaccine admixed with the Montanide ISA 720 VG adjuvant, iii) P + B using 5.0 μg of the vaccine admixed with the Montanide ISA 720 VG adjuvant, and iv) P + B of 50.0 μg of the vaccine admixed with the Montanide ISA 720 VG adjuvant (Figure 4D). The analyses conducted on blood samples collected every two weeks (over a total period of 6 weeks) revealed a dose- and time-dependent increase in antibody titer over time, and the differences observed between the medium (5.0 μg) and high (50.0 μg) doses were comparable overall (Figure 4E). Nevertheless, the vaccine tested at all doses did not elicit toxicity signs as depicted by the sustained and overlapping body weights collected over the entire immunization period (Figure 4F). Therefore, we can conclude from the results herein that aSp is compatible with different adjuvants and remains immunogenic even when tested in large, non-rodent animals.

### Clearance of the SARS-CoV-2 infection is faster in aSp-vaccinated hamsters

**Figure 5:**
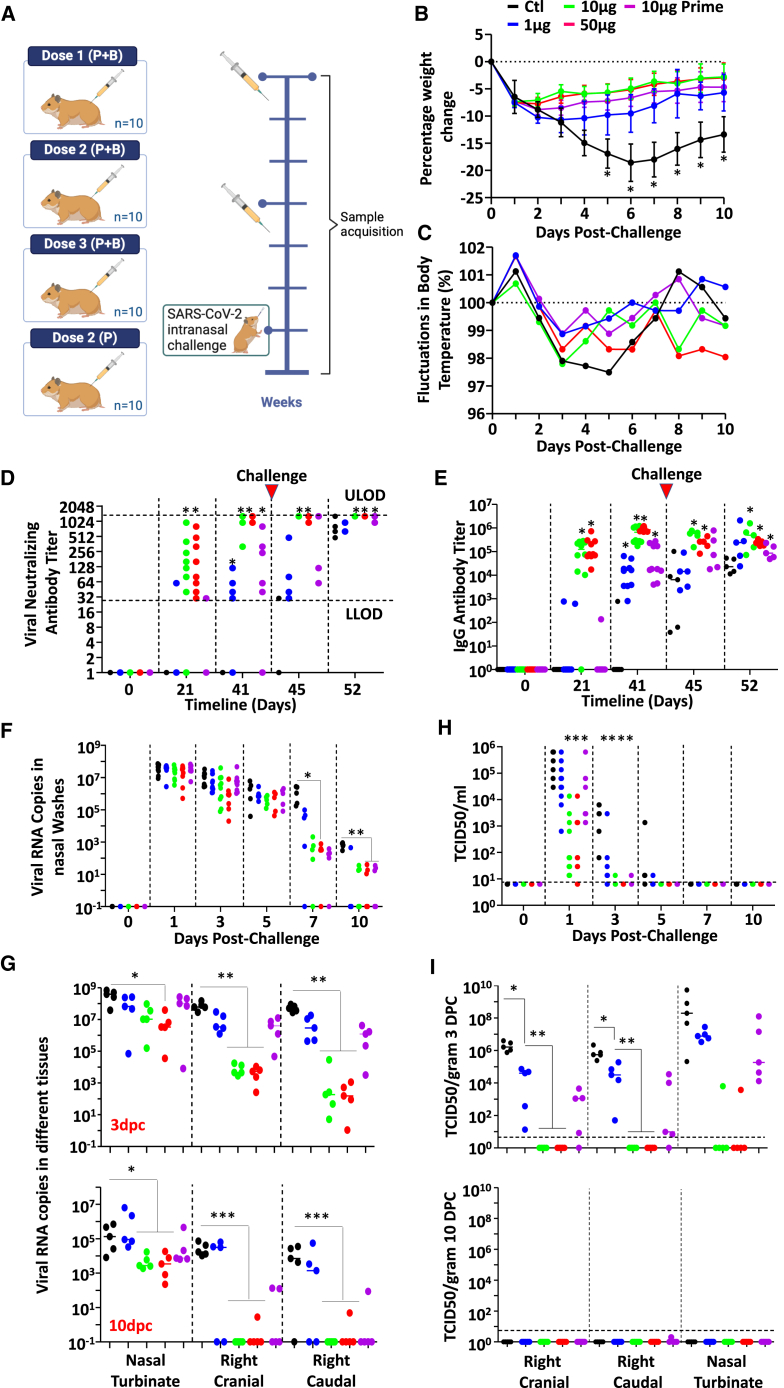
Vaccine immunogenicity and neutralizing the assessment of induced antibodies in a SARS-CoV-2 hamster model (A) Schematic representing the vaccination trial design conducted on hamsters. The groups are divided as follows: Dose 1 (P + B): 1 μg antigen; Dose 2 (P + B): 10 μg antigen; Dose 3 (P + B): 50 μg antigen; dose 2 (P): 10 μg antigen one dose only. Control hamsters received PBS. Immunizations were administered twice at a 21-day interval (Groups A, B, C, and D). Hamsters in Group E were immunized only once at day 21 with 10 μg antigen. Three weeks after the second immunization, all hamsters were intranasally challenged with the SARS-CoV-2 Wuhan strain. Hamsters were euthanized at either 3 or 10 dpc. (B) Body weight changes after challenge. For all subsequent experiments/panels, the placebo control group is shown in black, dose 1 in blue, dose 2 in green, dose 3 in red, and dose 2 (P) in purple. (C) Body temperature fluctuations after challenge. The color coding is the same as in panel B. (D) Titers of viral neutralization antibodies against the SARS-CoV-2 Wuhan strain. All hamsters were challenged on Day 42. The upper limit of quantification (ULOQ) was 1280, whereas the lower limit of detection (LLOD) was 20. Red arrow: virus challenge at Day 42. (E) Serum titers of antibody against the spike protein of SARS-CoV-2. Red arrow: virus challenge at Day 42. (F) Viral RNA levels in nasal washes after challenge. Spots on the x axis: Viral RNA is undetectable. (G) Viral RNA levels in tissues after challenge. Five hamsters per group were euthanized at 3 dpc, and the remaining hamsters were euthanized at 10 dpc. Spots on the x axis: Viral RNA is undetectable. (H) Infectious virus in nasal washes after SARS-CoV-2 Challenge. Horizontal dashed line: Lower limit of detection. (I) Infectious virus in tissues after SARS-CoV-2 Challenge. Horizontal dashed line: Lower limit of detection. For this experiment, *n* = 10/group, data are represented as mean ± SD with ∗*p* < 0.5, ∗∗*p* < 0.01 and ∗∗∗*p* < 0.001. Also see Figures S8 and S9.

Given the promising immunogenicity observed in aSp-immunized mice and rabbits, we next assessed the prophylactic potency of the vaccine in the “gold-standard” SARS-CoV-2 hamster model. The conducted trial consisted of administering a P + B at 1, 10 or 50 μg aSp as well as a single dose of 10 μg aSp all in combination with the Montanide ISA 720 VG adjuvant (Figure 5A). Although all vaccinated groups lost some weight following SARS-CoV-2 challenge, these losses were less pronounced compared to control animals, with a trend toward increased protection with escalating vaccine doses (Figure 5B; Figure S7). Analysis of temperature fluctuations, on the other hand, revealed no major deviations compared to control non-vaccinated animals. Temperature increased at one day post-challenge (dpc) and declined during the period of 2–7 dpc, which is normally seen in the hamster model (Figure 5C; Figure S8). With respect to the induction of neutralizing antibody titers, the P + B at 10 or 50 μg aSp induced significantly higher levels of both neutralizing (Figure 5D) and binding IgGs (Figure 5E) against the SARS-CoV-2 Sp compared to the placebo, 1 μg, or the single 10 μg dose groups. As for the presence of viral RNA in nasal washes, viral copies were similar across all groups at 1, 3-, and 5 dpc, but decreased rapidly in vaccinated animals on 7 and 10 dpc with many animals exhibiting undetectable levels, while all PBS complete animals had detectable viral RNA (Figure 5F). From a tissue localization perspective, the levels of infectious virus in nasal washes were significantly reduced in the groups receiving the P + B at 10 or 50 μg aSp compared to the other groups at both 3 and 10 dpc (Figure 5G). Similarly, the levels of viral RNA and infectious virus in nasal turbinate and lung tissues were significantly lower in the groups that received two doses of either 10 or 50 μg of aSp, with some animals again showing complete responses (Figure 5H–I). Altogether, our data demonstrates that vaccinating hamsters with a P + B regimen using 10 or 50 μg aSp reduces clinical signs and confers faster viral clearance following a SARS-CoV-2 infection.

### Vaccinated hamsters show no exacerbated signs of lung inflammation post-infection

**Figure 6:**
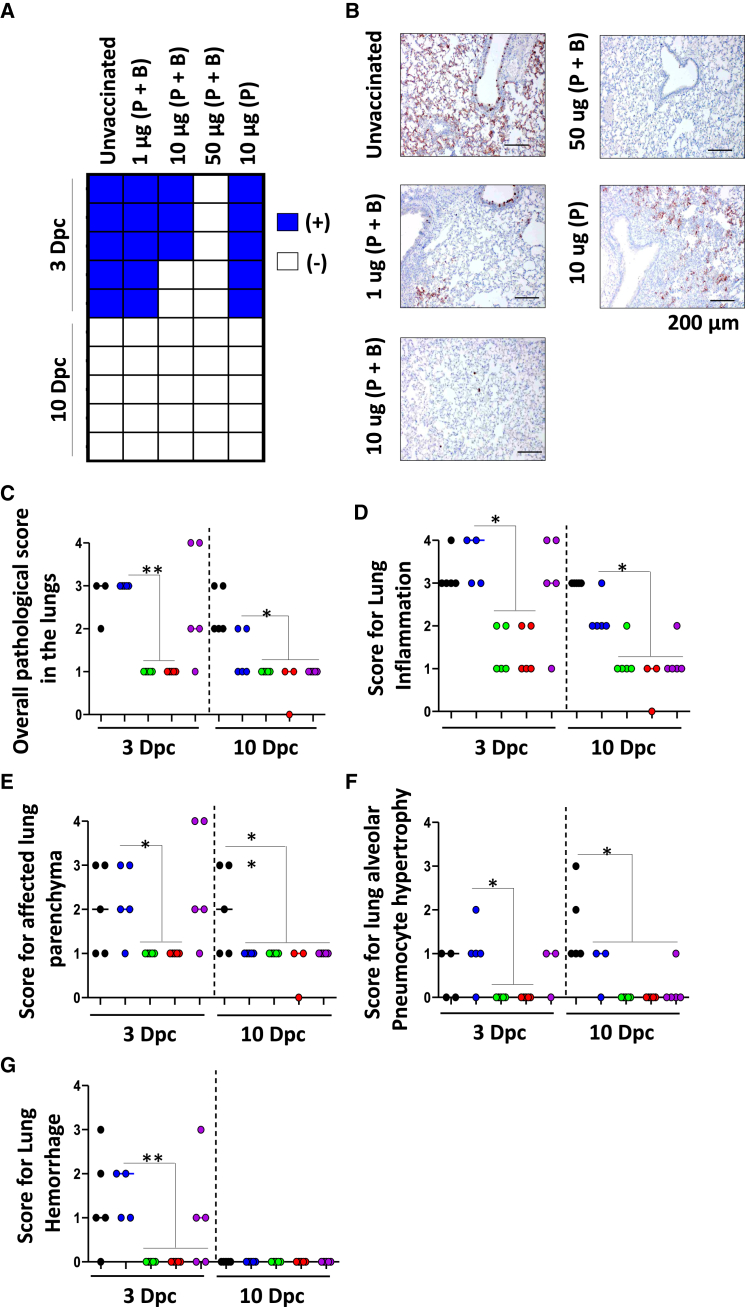
Immunohistochemical stain and histopathological alterations in hamster lungs after SARS-CoV-2 Challenge (A) A heatmap summarizing the presence (blue squares) or absence (empty squares) of SARS-CoV-2 antigen stain in the lungs of the 5 hamsters per group. (B) Representative picture of immunohistochemistry in each group. Scale bar represents 200 μm (C) Analysis of overall pathological scoring in the lungs for each group. (D) Scoring for lung inflammation at 3 and 10 dpc. (E) Scoring for affected lung parenchyma at 3 and 10 dpc. (F) Scoring for lung alveolar pneumocyte hypertrophy at 3 and 10 dpc. (G) Scoring for lung hemorrhage was conducted at 3 and 10 dpc. For panels C and D, the severity of inflammation in the lungs was scored as follows: 0 for inflammation absent; 1 for minimal inflammation; 2 for mild inflammation; 3 for moderate inflammation; and 4 for marked inflammation. For all remaining scoring, a 0 was given to absent events; 1 for events of 1–24%; 2 for events of 25–49%; 3 for events of 50–74%, and 4 for events of 75–100%. Data are represented as mean ± SD with ∗*p* < 0.5, ∗∗*p* < 0.01 and ∗∗∗*p* < 0.001. Also see Figures S10–S12.

Based upon our data from vaccinated hamsters, we next conducted an in-depth histological investigation for the presence of inflammatory signs in the lungs of these animals. When assessed at 3 dpc for the presence of viral SARS-CoV-2 antigen, we observed no symptoms in any hamsters immunized with 50 μg of aSp, whereas the remaining groups had detectable viral antigen (Figures 6A and 6B). At 10 dpc, viral antigen was undetectable in the lungs of any animals (Figure 6A). Interestingly, hamsters undergoing a P + B regimen using the 10 or 50 μg aSp show lung pathological alterations with scores between 0 and 1 (Figure 6C), or an average score of 1–2 for lung inflammation (Figure 6D; Figure S9A). In addition, both areas of parenchyma affected (Figure 6E; Figure S9B) and hypertrophy of alveolar pneumocytes in the lungs of these animals revealed milder pathological signs (Figure 6F; Figure S10A). Moreover, there was no evidence of hemorrhage in any of these vaccinated animals (Figure 6G; Figure S10B). In contrast, nearly all hamsters in the placebo, 1 μg P + B, or the single 10 μg dose groups had moderate to severe lung histopathology with scores of 2–4 (Figures 6C–6G). The pathology data correlated well with an independent measure of lung to body weight ratios at both 3 and 10 dpc (Figures S11 and S12). Taken together, a P + B regimen using 10 or 50 μg of the vaccine showed evidence of a high level of protection against SARS-CoV-2 challenge in hamsters with no apparent exacerbated lung toxicity.

## Discussion

The Accum technology was initially designed to disrupt the integrity of endosomal membranes in order to enhance the bioaccumulation of chemotherapeutic agents delivered via antibody-drug conjugates.^34^^,^^36^^,^^38^ However, we recently demonstrated that the same platform could also be used in the design of cell- and protein-based vaccines.^35^^,^^39^^,^^40^ For instance, when dendritic cells were pulsed with ovalbumin or tumor lysate conjugated to Accum, the antitumoral activity triggered by this cellular vaccine was substantially enhanced, resulting in partial and complete responses in tumor-bearing mice.^39^ Likewise, vaccination using an Accum-E7 oncoprotein conjugate elicited a powerful anti-tumoral response capable of impairing cervical cancer growth.^40^ Although both studies were centered on cellular immunity (e.g., focused on CD8 T cell responses), animals vaccinated using both experimental vaccines elicited mixed immune responses as antibodies were also detected in the sera of vaccinated animals.^39^^,^^40^

Based on our unexpected observations above, we next elected to investigate whether Accum-induced humoral responses can be harnessed in the context of infectious diseases such as SARS-CoV-2. The administration of aSp not only elicited strong antibody titers in immunocompetent mice, but these antibodies were mostly directed toward the S1 domain of Sp and cross-reacted with different RBD variants. Furthermore, aSp was shown to be safe and immunogenic in rabbits, an observation that was further replicated in the “gold-standard” hamster SARS-CoV-2 model in which aSp administration resulted in faster viral particle clearance from the respiratory tract following a challenge with the Wuhan strain. Surprisingly, the hamster group vaccinated with the highest aSp dose showed no exacerbated lung inflammatory reactions, which are often observed in infected hosts (see graphical abstract - GA).^49^^,^^50^

The higher antibody titer detected following aSp vaccination may suggest potential and unexpected alterations occurring at the protein level, such as structural stabilization and/or exposition of certain immunogenic peptides. Our structural analyses (i.e., cryo-EM), which directly links to 3D/quaternary structure, refuted that hypothesis as no changes were observed in the overall quaternary structure of the protein. This indicates that the observed therapeutic potency in animals may be related to the native properties of the Accum moiety itself, which begs the question: how could a molecule designed to trigger endosome-to-cytosol escape trigger a strong and effective humoral response? The answer to this question may rely on the basics of antigen presentation. In general, endogenous antigens normally present in the cytosol are digested by the proteasomal complex, resulting in the production of 8–11 amino acid-long peptides suitable for MHCI presentation to CD8 T cells. On the other hand, engulfed exogenous antigens are degraded in endosomes and/or lysosomes by specific proteases to generate longer peptide fragments, which are then presented by MHCII to CD4 T cells.^35^^,^^39^^,^^40^ While this paradigm is true in general, alternative pathways have been described where cytoplasmic or nuclear proteins can result in MHCII presentation.^51^ In fact, proteins can transit from the cytosol to lysosomes during autophagy, which has been reported to occur in antigen-presenting cells in response to starvation and/or internal structural damage.^52^ In microautophagy, for example, small portions of the cytosol are internalized through lysosomal invaginations.^53^^,^^54^^,^^55^ Macroautophagy, on the other hand, is a cellular process aiming at the degradation of long-lived proteins and organelles.^52^ Lastly, under specific conditions, the process of chaperone-mediated autophagy can be activated, where a complex of multiple heat-shock proteins can transport substrate/aggregated proteins to the lysosomal membrane. These proteins can then interact with lysosome-associated membrane protein-2, resulting in their translocation across the lysosomal membrane.^56^ Although autophagy is normally triggered under starvation or nutrient deprivation, this cellular process may intersect with the endocytic pathway when structural damages such as Accum-induced endosomal membrane breaks occur.^57^ Although investigating autophagy is beyond the scope of the present study, Accum-mediated transport of damaged endosome-containing captured antigens to lysosomes may be a plausible explanation for the induced humoral response. Besides, multiple other factors such as the type of antigen-presenting cell affected, protein half-life and subcellular distribution may influence which transport pathway is utilized. Thus, future studies focused on better clarifying the mode of action of Accum in the context of humoral immunity are needed.

Although mRNA-based SARS-CoV-2 vaccines have been undeniably crucial in combating the COVID-19 pandemic and restoring society to “normal” function in a safe and efficacious manner, the temperature-sensitive fragility of mRNA vaccines remains a major logistical hurdle (i.e., transport and storage) to overcome during crucial mass vaccination campaigns.^21^ In parallel, the results from our present study highlight the promising application of the Accum technology for the future development of prophylactic vaccines that are protein-based given that the target antigen is identified and manufactured.

### Limitations of the study

The main goal of the herein study was to investigate the humoral response induced by the aSp vaccine in multiple animal models as a proof-of-concept for the use of Accum in the rapid and effective design of protein-based vaccines for future pandemics. However, limitations of this study include two main points. First, a more in-depth mechanistic analysis deciphering how Accum bioconjugation enhances humoral immunity is warranted. As such, future studies focusing on the potential induction of autophagy or other autophagy-related mechanisms that may be induced in response to damaged endosomal structures are required. This could then highlight the fact that Accum-bioconjugated proteins could, on one end, escape the endosome and trigger potent CD8 T cell response, while some structures are re-sorted down “specific recycling” pathways as a defense mechanism to repair damaged structures. Second, our study was mainly centered around humoral responses with no investigations conducted on the CD8 T cell response, which are most likely induced in response to aSp vaccination and play important roles in clearing virally infected cells. This may perhaps explain the strong IFN-γ production and the faster clearance of the viral load in the nasal cavity of infected hamsters, along with the absence of lung inflammation.

## Resource availability

### Lead contact

Further information and requests for resources and reagents should be directed to and will be fulfilled by the Lead contact, Moutih Rafei (moutih.rafei.1@umontreal.ca).

### Materials availability

Some reagents used in this study can be made available on request. This might require a payment and/or a Materials Transfer Agreement if there is potential for commercial application.

### Data and code availability

• The map and model for the aSp cryo-EM have been deposited in the EMDB (EMD-45411) and PDB (9CB0), respectively.
• This article does not report original code. Any analyses applied are based on previously available software, primarily Maestro, GraphPad, and cryoSPARC.
• Any additional information required to reanalyze the data reported in this article is available from the lead contact upon request.

## Acknowledgments

We wish to thank all technicians from the Université de Montréal IRIC Animal Facility for their kind support regarding some of the murine *in vivo* experiments, as well as Dr. Eric Bonneil from the IRIC Proteomics Facility for access to his mass spectrometry expertise. We thank Claire Atkinson and Natalie Strynadka from the High-Resolution Macromolecular Electron Microscopy (HRMEM) facility at the University of British Columbia (https://cryoem.med.ubc.ca), for access to the microscope facility used in this work. HRMEM is funded by the Canadian Foundation for Innovation and the British Columbia Knowledge Development Fund. The McGill SPR-MS Facility thanks the Canada Foundation for Innovation for infrastructure support. Some of the figures displayed in the article were generated using the Biorender illustration software. GAM is a recipient of a postdoctoral fellowship from the Natural Sciences and Engineering Research Council of Canada of Canada. RF is the recipient of a PhD award from the Cole Foundation. NAC is supported by a Transition Award from the Center for Blood Research. VIDO receives operational funding from the Canada Foundation for Innovation through the Major Science Initiatives, from the Government of Saskatchewan through Innovation Saskatchewan, and the Ministry of Agriculture. This study was supported by a contract research grant (#RB002835) from Defence Therapeutics Inc.

## Author contributions

JPB conducted most of the *in vitro* antibody analysis. NAC worked on the Cryo-EM data. NEH worked on the protein structure. GAM and JAC analyzed the CD and fluorescence spectroscopy results. DS and SB performed the Accum bioconjugations. JA, RF, and MDG contributed to data analyses and generated schematic diagrams. MAH performed all SPR data acquisition and analyses. DF, ML, GH, and QL conducted, analyzed and wrote the article sections related to the *in vivo* work conducted on hamsters. ST contributed to the study design. MR conceived and supervised the project, analyzed all collected data, and wrote the first draft of the article. All authors contributed to article editing.

## Declaration of interests

Daniela Stanga, Marina P. Gonçalves, and Simon Beaudoin were employees of Defense Therapeutics Inc. at the time of the study and declare competing financial interests. All remaining authors declare no competing interests.

## STAR★Methods

### Key resources table

**Table:** 

REAGENT or RESOURCE	SOURCE	IDENTIFIER
**Antibodies**		

Goat Anti-Mouse IgG HRP	R&D systems	Cat#: HAF007; RRID:AB_357234
Goat anti-Syrian Hamster IgG (H + L)	Invitrogen	Cat#: PA1-29626; RRID:AB_10985385
Mouse anti-SARS-CoV-2 spike	NOVUS	Cat#: NBP3-06856; RRID:AB_3553650
Mouse anti-SARS-CoV-2 spike	NOVUS	Cat#: NBP3-12855; RRID:AB_3587576
Mouse anti-SARS-CoV-2 spike	NOVUS	Cat#: NBP3-06857; RRID:AB_3553651

**Chemicals, peptides, and recombinant proteins**		

3,3′,5,5′-Tetramethylbenzidine (TMB)	Sigma Aldrich	Cat#: T0440
AddaS03™	InvivoGene	Cat#: vac-as03-10
AddaVax™	InvivoGene	Cat#: vac-adx-10
ONE-Glo™ EX Luciferase Assay Substrate	Promrga	Cat#: E8110
OPD Substrate Tablets (*o*-phenylenediamine dihydrochloride)	ThermoFisher Scientific ™	Cat#: 34006
Pierce™ Gentle Ag/Ab Elution Buffer, pH 6.6	ThermoFisher Scientific ™	Cat#: 21027
Recombinant human ACE2	Creative Biomart	Cat#: ACE2-736H
SARS-CoV-2 (COVID-19) S protein RBD	ACRO Biosystems	Cat#: SPD-C52H2
SARS-CoV-2 (COVID-19) S protein RBD (K417N, E484K, N501Y)	ACRO Biosystems	Cat#: SPD-C52Hp
SARS-CoV-2 (COVID-19) S protein RBD (L452R)	ACRO Biosystems	Cat#: SPD-C52He
SARS-CoV-2 (COVID-19) S protein RBD (N501Y)	ACRO Biosystems	Cat#: SPD-C52Hn
SARS-CoV-2 S protein RBD (K417T, E484K, N501Y)	ACRO Biosystems	Cat#: SPD-C52Hr
SARS-CoV-2 Sp protein	ACRO Biosystems	Cat#: SPN-C52H9
SARS-CoV-2 Spike RBD Protein (L452R, E484Q)	ACRO Biosystems	Cat#: SPD-C52Hv
SEPPIC INC MONTANIDE ISA-720 VG ST	SEPPIC	Cat#:NC0685296
Sequencing Grade Modified Trypsin	Promega	Cat#: V5111; Lot#: 0000555098
Sulfo-SMCC (sulfosuccinimidyl 4-(N-maleimidomethyl)cyclohexane-1-carboxylate)	ThermoFisher Scientific ™	Cat#: 22322
Tween 20 ®	Amresco	Cat# 0777-1L

**Deposited data**		

Electron Microscopy DataBank	EMDB	45411
Protein Bank Data	PDB	9CB0

**Experimental models: Organisms/strains**		

Mouse: C57BL/6	The Jackson Laboratory	Cat#: 000664
New Zeland female rabbits	Charles River	Cat#: 052
Syrian Hamster	Charles River	Cat#: 049

**Software and algorithms**		

cryoSPARC	https://cryosparc.com/	CryoSPARC v4.7.0
GraphPad Prism	https://www.graphpad.com	Version 10.4.2
Maestro	https://www.schrodinger.com/platform/products/maestro/	Version 11.6. Release 2018

**Other**		

96-well microtiter Nunc MacIvor plates	ThermoFisher Scientific™	Cat#: 442404

### Experimental model and study participant details

#### Animal and ethics approval

All female C57BL/6 mice (6–8 weeks old) were purchased from the Jackson Laboratory (Bar Harbor, ME, USA). Animals were housed in a pathogen-free environment at the animal facility located at the Institute for Research in Immunology and Cancer (IRIC). All experimental procedures were approved by the Animal Ethics Committee of Université de Montréal (#22–065). Twelve 4 months-old female rabbits were purchased from Charles River Canada Inc. (Saint Constant, QC, Canada). The study plan for the rabbit -related work was reviewed and assessed by the Animal Care Committee (ACC) of ITR Laboratories (Study #700677B). All 5-6-week-old male Syrian golden hamsters were obtained from Charles River Laboratories (Saint Constant, QC, Canada). The study plan for the hamster-related work was reviewed and assessed by the Animal Research Ethics Board (AREB) of the University of Saskatchewan (study# AS21-048IV).

#### Immunization of immunocompetent C57BL/6 mice

Female 8–10 weeks old C57BL/6 mice (*n* = 6/group) were used in the immunization study. Briefly, a 10 μg of Sp or aSp was mixed with AddaVAX or AddaSO3 in a 1:1 v/v ratio according to manufacturer’s instructions. The vaccine was then injected subcutaneously at two distal sites on day 0 and 14. Pre-immune sera was collected from all animals at day −3. A recall vaccination was conducted again at week 17 to re-boost the humoral response.

#### Dose toxicity study (New Zealand female rabbits)

The test and control items were prepared, on each day of dose administration and were administered to groups of female rabbits (*n* = 3/condition) on Days 0 and 14 by subcutaneous injection administration. All mixing steps were performed using clean techniques under a laminar flow hood. All formulations were prepared by mixing appropriate volumes of the solutions, to the adjuvant at a ratio of 3:7, using 2 syringes connected via a connector. Once properly mixed, individual syringes containing the appropriate volumes to be administered for each animal were prepared and were appropriately identified. At the initiation of treatment (Day 1), rabbits were approximately 4 months old, and their body weights ranged from 3.6 to 4.1 kg. Blood samples (approximately 1 mL each) were collected from all animals on Days 0 (prior to dose administration), 14 (prior to dose administration), 28, and 42. For this purpose, rabbits were bled via the central ear artery and samples were collected into tubes containing clotting activator. Following collection, blood samples were allowed to stand at room temperature for a minimum of 30 minutes to ensure complete clotting, then samples were centrifuged (2500 rpm for 10 minutes at approximately 4°C) and the resulting serum recovered, split into 2 aliquots (set 1 and set 2) of approximately the same volume in labeled tubes, and placed on dry ice pending storage in a freezer (≤−60°C). Set 2 samples were retained at ITR. Additional parameters monitored during this study included mortality, clinical observations, body weights, food consumption, skin evaluation, clinical pathology evaluation, as well as any pathology examination. Antibody levels noted at ≥ 5 μg/dose were considered dose- and time-dependent, with a powerful antibody response and a plateau reached at Day 28. The subcutaneous injection of aSp to female rabbits on Days 0 and 14 resulted in signs of local irritation noted clinically (edema and erythema) at ≥ 0.5 μg/dose at dosing site, correlating with microscopic findings at dosing sites, including edema and cell infiltrate at all dose levels and necrosis at 50 μg/dose. No sign of systemic toxicity was noted at ≤ 5 μg/dose, but mineralization of the renal cortex was noted at 50 μg/dose. Since none of these observations were considered adverse, the No Observed Adverse Effect Level (NOAEL) is considered to be 50 μg/dose.

#### Immunization and challenge (hamster model)

Five groups of 5-6-week-old male Syrian golden hamsters (*n* = 10/group) were used in this study. Animals were randomly assigned to groups, and animal personnel were blinded to the treatment. One day prior to immunization hamsters were anesthetized and implanted with a transponder chip (BioMedic Data Systems) for ID and temperature monitoring. On day 0, hamsters were immunized with 1 mL of vaccine via the subcutaneous route with placebo (vehicle only), or vaccines containing 1, 10 or 50 μg of antigen as indicated. Immunizations were administered in a 21-day prime/boost (P + B) interval. An additional group received only a single immunization on day 21 with 10 μg antigen. Three weeks following the second immunization, all hamsters were challenged via the intranasal route (50μL/nare) with a total of 1 × 10^5^ tissue culture infectious dose (TCID)_50_ of SARS-CoV2/Canada/ON/VIDO-01/2020/Vero’76/p.2, an ancestral B lineage isolate (sequence available at GISAID-EPI_ISL_425177). The challenge portion of the study was performed in a containment level 3-Ag laboratory at VIDO, according to approved protocols and standard operating procedures. Blood was collected in serum separator tubes (SST) prior to immunization (days 0 and 21), prior to challenge (day 41) and at euthanasia (either day 45 or 52). Nasal washes were collected 1 day prior to challenge and every second dpc. Five hamsters per group were euthanized at either 3 dpc or 10 dpc as indicated. At necropsy, the left lung lobe was fixed with 10% neutral buffered formalin for histopathology (H&E stain). The right cranial and right caudal lung lobes, as well as the nasal turbinates, were collected and placed in RNAlater (Qiagen) for viral load by RT-qPCR and into Dulbecco’s Modified Eagle Medium (DMEM) for levels of infectious virus by cell culture TCID_50_.

#### Immunohisotochemical and histopathology analysis of lungs tissues (Hamster model)

At necropsy, the left lung of vaccinated hamsters were placed into 10% neutral buffered formalin to fix the tissues. Tissues were transferred to Prairie Diagnostic Services (Saskatoon, SK), where they were embedded, sectioned and stained with hematoxylin and eosin (H&E). Slides were then scored by in a blinded manner by a board-certified pathologist according to an established scoring system for SARS-CoV-2. Sections of lung tissues were also prepared for immunohistochemical staining, which was conducted at Prairie Diagnostic Services using an automated slide stainer (Autostainer Plus, Agilent Technologies Canada Inc., Mississauga, ON). Epitope retrieval was performed in a Tris/EDTA pH 9 buffer at 97°C for 20 minutes. The primary antibody was an in-house generated rabbit polyclonal antibody against the nucleocapsid protein of SARS-CoV-2 (SARS2-N of the Wuhan strain). The SARS2-N antibody was diluted 1:800 in PBS and incubated with the slides for 30 min at room temperature. After washing, the bound SARS2-N antibody was then detected using an HRP-labelled polymer detection reagent (EnVision+ System - HRP Labeled Polymer, Agilent Technologies Canada Inc., Mississauga, ON). Immunostaining was categorized as no stain, weak stain intensity, or strong stain intensity.

### Method details

#### Animal-derived sera analysis by ELISA

A 100 μL volume at 1 μg/mL Sp protein (Acro Biosystems; Cat#: SPN-C52H3) was diluted in PBS pH 7.4 prior to coating a 96-well microtiter plates with overnight incubation at 4°C. The following day, the coating solution was discarded and plates blocked with 150 μL of PBS containing 3% skim milk for 1 h hour at room temperature. Using U-bottom Microplate or Eppendorf tubes, sera samples were diluted 1:100 followed by two-fold dilution series in PBS containing 1.5% skim milk and 0.05% Tween 20. All blocked plates are washed 3X with 400 μL/well PBS-T (PBS containing 0.05% Tween 20) before 100 μL diluted sera was then added to the wells and incubated for 2 hours at room temperature. Plates are washed 3X with 400 μL/well PBS-T before adding 100 μL/well of anti-mouse IgG conjugated HRP antibody diluted 1:1000 in PBS containing 1.5% skim milk and 0.05% Tween 20. After 2 hours at room temperature, the plates were washed 3X with 400μL/well PBS-T before adding 100μL/well TMB substrate for signal detection. At the end of the incubation period (20 min at room temperature), 50 μL of 1M H_2_SO_4_ was added per well to stop the development reaction. Optical density was then measured using a microplate reader set to wavelengths 450 nm (signal) and 570 nm.^58^ The same ELISA was repeated using the three commercially available mAbs (clone B3476M; clone B3478M; and clone 1C3H9). Both Sp and aSp proteins were tested.

#### ELISA analyses: Hamster model

96-well plates were coated with 1 μg/mL of SARS-CoV-2 S antigen (Acro Biosystems; Cat# SPN-C52H3, Lt# 3841-208DF1-W8) in PBS. Plates were blocked with 5% skim milk powder in PBS-T (PBS containing 0.05% Tween 20). Serum was diluted in PBS starting at 1:100 followed by 4-fold serial dilutions. Diluted samples were plated in duplicate. Goat anti-hamster IgG HRP was used as the secondary antibody at 1:7000. Throughout the assay, plates were washed with PBS-T. Plates were developed with OPD peroxidase substrate (0.5 mg/mL). The reaction was stopped with 2.5 M sulfuric acid and absorbance was measured at 490 nm. The cutoff value was determined by taking the average of the negative controls (negative sera) plus 3 standard deviations. To calculate the titers, the forecast function in an intercept program built in Excel was used to generate the intercept values, followed by manual assignment of “1” for any values below the cut-off value.

#### Virus neutralization assays using hamster-derived sera: Hamster model

Viral neutralization (VN) assays using the indicated SARS-CoV-2 isolate were performed on the serum samples using Vero’76 cells. Serum was heat-inactivated for 30 min at 56°C, then serially diluted (2-fold). The experiment was conducted in technical duplicates. The indicated virus was diluted in medium to a concentration of 25 TCID_50_ per 50 μL per well (the inoculum size = 25 TCID_50_). Next, 60 μL of the virus solution was mixed with 60 μL serially diluted serum samples. The mixture was incubated for 1 h at 37°C with 5% CO_2_. The pre-incubated virus-serum mixtures (100 μL) described above were transferred to the wells of the 96-well flat-bottom plates containing 90% confluent pre-seeded Vero’76 cells. The plates were incubated at 37°C, with 5% CO_2_ for five days. The plates were observed using a microscope 1 day post-infection (dpi) for contamination and on 3 and 5 dpi for cytopathic effect. The serum dilution factor for the wells with no cytopathic effect (CPE) at 5 dpi was defined as the serum neutralization titer. The initial serum dilution factor was 1:20 (the lower limit of detection). The highest dilution of the sera was 1280 (the upper limit of quantification).

#### Pseudo-particle neutralization assay

The neutralizing capacity of collected sera was assessed using a cell-based Pseudo-particle Neutralization Assay (the assay was performed by Nexelis, Laval, QC, Canada). Vero E6 cells expressing the ACE-2 receptor were seeded in 96-well white plates at 20 000 cells/well to reach a cell confluence of 80%. Serum samples and controls were diluted in duplicates in cell growth media at a starting dilution of 1/25, 1/250 or 1/2500 (for mouse samples) followed by a serial dilution (2-fold dilutions, 6 times). In parallel, SARS-CoV-2 pseudovirus (lots NL2011Q-N) was diluted as to reach the desired concentration (according to pre-determined TU/mL). Pseudovirus was then added to diluted serum samples and pre-incubated for 1 hour at 37°C with 5% CO2. The mixture was then added to the pre-seeded Vero E6 cell layers and plates were incubated for 18–20 hours at 37°C with 5% CO2. Following incubation and removal of media, ONE-Glo EX Luciferase Assay Substrate was added to cells and incubated for 3 minutes at room temperature with shaking. Luminescence was measured using a SpectraMax iD3 microplate reader and SoftMax Prov7.01 (Molecular Devices, San Jose, CA). Luminescence results for each dilution were used to generate a titration curve using a 4-parameter logistic regression (4 PL). A titer for each replicate was defined as the reciprocal dilution of the sample for which the luminescence is equal to a pre-determined cut-point of 50, corresponding to 50% neutralization. This cut-point was established using linear regression using 50% flanking points. The final titer was calculated as the average of replicates.

#### Accum® synthesis

The Accum® molecule was synthesized by CanPeptide Inc. as previously reported.^39^ All chemicals, resin and solvents were used as received from suppliers. Fmoc-protected amino acids, diisopropylethylamine (DIPEA), 2-(1H-7-Azabenzotriazol-1-yl)-1,1,3,3-tetramethyl uronium hexafluorophosphate methanaminium (HATU) and trifluoroacetic acid (TFA) were purchased from Chem-impex international (Wood Dale, IL). The rink amide resin was obtained from Rapp Polymere (Tübingen, Germany). ChAc, triisopropylsilane (TIPS) and ethanedithiol (EDT), were obtained from Sigma-Aldrich (St-Louis, MO). Dimethylformamide (DMF), isopropanol (IPA) and dichloromethane (DCM) were purchased from VWR (Québec, Canada). Piperidine was obtained from A&C Chemicals (Québec, Canada). UPLC-MS analyses were performed with a Waters (Milford, MA) AQUITY H-class – SQD2 mass detector and PDA eλ UV-visible detector on a BEH, C18, 1.7 μm, 2.1 × 50 mm. Purifications were performed on a Waters preparative UPLC system consisting of injector 2707, pump 2535, and detector 2489, with an ACE C18 column 250 × 21.2 mm, 5 μm (Canadian Life Science, Ontario, Canada). For analytical UPLC, water and acetonitrile with 0.1% formic acid were used. For preparative UPLC, water plus 0.1% TFA, and pure acetonitrile were used. Peptide syntheses were performed on Tribute UV-IR automated peptide synthesizer from Protein Technologies (Tucson, AZ) following manufacturer’s recommendations. Peptides were synthetized on the solid phase Rink Amide resin (loading 0.22 mmol/g) using an automated Tribute UV-IR Peptide Synthesizer, at 50 μmol scale. Fmoc groups deprotection was achieved using 20% piperidine in DMF using the UV monitoring smart deprotection feature. Couplings were performed using 5 eq of amino acids, activated with HATU and DIPEA (1:2 M ratio in relation to the amino acid) for 2 minutes with IR heating at 50°C (except for Fmoc-Cys (Trt), 20 minutes at room temperature). The final deprotection was performed manually using 50% piperidine in DMF for 30 minutes and resin were washed using DMF x2, DCM x3, and IPA. The ChAc unit was coupled using 5 eq of the acid, activated with HATU and DIPEA (1:2 M ratio in relation to the ChAc) for 16 hours and then resin was washed as described above. The peptides were cleaved from their solid support using a mixture of TFA/H2O/TIPS/EDT (92.5/2.5/2.5/2.5) (4 mL for 200 mg of resin) for 3 hours. Crude peptides were precipitated in chilled diethyl ether, centrifuged, and allowed to dry prior to reverse phase preparative UPLC purification. Final peptides were characterized using mass spectroscopy and UPLC.

#### Sp protein bioconjugation to Accum®

The Sp protein conjugation to Accum® was performed using a Sulfo-SMCC linker to target exposed lysine residues on the protein. Briefly, Sulfo-SMCC was added in a 10- or 50-fold molar excess to the Sp protein and incubated for 1 hour at room temperature in PBS, pH 7.6. The maleimide-derivatized protein was then purified using Amicon® Ultra centrifugal filters (100 kDa cutoff) and reacted with a 100-fold molar excess of Accum® overnight at 4°C, followed by purification using the same Amicon filters. To evaluate the success of bioconjugation, the unmodified (Sp) and modified (aSp) proteins were analyzed by SDS-PAGE (5 μg protein per lane; 4–12% gradient gel under reducing conditions; Coomassie blue staining).

#### CD spectroscopy

The CD spectroscopy was performed on a Jasco J-810 spectropolarimeter outfitted with a Peltier-type thermostat scanning at 0.1 nm resolution and averaged over 10 scans. Temperature denaturation experiments were performed at a heating rate of 1 °C/min. All samples were measured at a concentration of 2 μM (Sp or aSp) in a 10 mM sodium phosphate buffer pH 7.4 (unless otherwise stated) sodium phosphate buffer (10 mM) using a 1.0 mm path length quartz cuvette. Chemical denaturation experiments were performed using TCEP as a denaturant and analyzed under the abovementioned conditions. The quantification of secondary structural components was performed using the BeStSel tool.

#### Fluorescence spectroscopy

Fluorescence spectroscopy was performed using a using a Hitachi F-2500 FL spectrophotometer at a concentration of 2 μM protein (Sp or aSp) in a 10 mM PBS pH 7.4 (unless otherwise stated) using a 1 cm path length quartz cuvette. Scans were performed at 100 nm/min at a resolution of 0.5 nm, excitation and emission slits were set to 5.0 nm. Temperature dependent measurements were collected in 5°C intervals, the protein solution was stirred for 3 minutes at each temperature prior to spectroscopic analysis.

#### Mass spectrometry

To identify the number of Accum® molecules on Sars-Cov-2 Spike Protein by LC-MS, aSp samples were digested with 1 μg sequencing grade trypsin overnight at 37°C on a Thermo mixer at 500 rpm without reduction and alkylation. Peptides were separated on a home-made reversed-phase column (150-μm i.d. by 200 mm) with a 56-min gradient from 10 to 30% ACN-0.2% FA and a 600-nL/min flow rate on an Easy nLC-1200 connected to an Orbitrap Exploris 480 (Thermo Fisher Scientific, San Jose, CA) with the following MS parameters: m/z range was 350–1200; MS Resolution was set at 120K with 50 ms injection time. MS/MS parameters were as follows: Intensity Threshold 60000, charge state 2–4, dynamic exclusion 60s, MS/MS Resolution 30K, Injection Time 75s, Normalized collision energy 34%. Raw files were analyzed with MaxQuant (version 2.0.3.0). A customized database was made with the antibody sequence. Mass tolerances on precursor and fragment ions were 10 ppm and 0.01 Da, respectively. Fixed modification was carbamidomethyl (C). Variable selected posttranslational modifications, oxidation (M) and deamidation (NQ). FDRs for peptides and cross-linked peptides were set to 1%. The drug-antibody ratio (DAR) was calculated following the method developed and detailed by Wang et al. and Li et al. for digested antibody-drug conjugates.^59^ Briefly, the conjugation level of each sequence is calculated using the weighted average of the extracted ion peak area (called intensity in Table S1). Taking into consideration that the Sp protein has a trimeric structure rather than the heavy and light chains of an antibody, the average DAR was calculated as three times the summed conjugation level for each site.

#### Cryo-EM sample preparation

Aliquots of 3 μL of aSp were applied to glow-discharged Quantifoil® (1.2/1.3) grids. The grids were blotted for 12 seconds at 100% humidity with an offset of −10 and plunged frozen into liquid ethane using a Vitrobot Mark IV (Thermo Fisher). Grid screening and dataset collection occurred at UBC HRMEM. Grids were imaged on a 300 keV Titan Krios microscope equipped with a Falcon IV camera and an energy filter (Thermo Fisher). Movies were collected at a magnification corresponding to a 0.77 Å per physical pixel. The dose was set to a total of 50 electrons per Å^2^. Automated data collection was carried out using EPU (Thermo Fisher) with a nominal defocus range set from −0.8 to −2.0 μM.

#### Surface plasmon resonance (SPR) binding specificity

Binding between ACE2 (recombinant human ACE2, ∼105 kDa; Creative Biomart #ACE2-736H) and the Spike protein (Sp; SARS-CoV-2 S protein (D614G) super stable trimer, ∼185 kDa; Acro Biosystems #SPN-C52H3) was monitored using a BIACORE 3000 system (Cytiva Life Sciences). SPR experiments were performed on research-grade CM5 sensor chips at 25°C using filtered (0.2 μm) and degassed HBS-ZT running buffer (10 mM HEPES pH 7.4, 150 mM NaCl, 10 μM ZnCl_2_, 0.05% (v/v) Tween 20). Immobilized protein surfaces were prepared using the Biacore Amine Coupling Kit as recommended by the manufacturer (10 μg/mL ACE2 in 10 mM sodium acetate pH 4.5 containing 10 μM ZnCl_2_, or 10 μg/mL Sp in 10 mM sodium acetate pH 5.5; each 800–1300 RU final density); corresponding reference surfaces were prepared in the absence of protein. Protein-grade detergents (Tween 20 and Empigen) were from Anatrace (Maumee, OH, USA) and Pierce Gentle Ag/Ab Elution Buffer pH 6.6 (PGE) was from Thermo Fisher Scientific (catalog #21013); all other reagents were of analytical grade quality. To assess binding specificity, fixed injections (250 nM) of buffer (negative), human serum albumin (HSA), or ACE2 were flowed over reference and protein-immobilized surfaces at 25 μL/min in ‘KINJECT’ mode (2 min association ±5 min dissociation). Between sample injections, surfaces were regenerated at 50 μL/min using sequential 30 s pulses of solutions I (3M MgCl2 containing 0.1% (v/v) Empigen), II (0.2 M Tris-HCl pH 8.0 containing 1 M NaCl and 0.1% (v/v) Empigen), and III (1:1:1 (v/v) mix of PGE, 100 mM H3PO_4_, and 1 M NaCl).

#### SPR binding kinetics and affinity

ACE2 (0–250 nM; 2-fold dilution series) was titrated over reference and Sp-immobilized surfaces at 25 μL/min in multi-cycle mode (5 min association +15 min dissociation) and regenerated as above. To assess the anti-Spike protein neutralization abilities of generated antibodies, minimally diluted sera samples #1–5 (only 1/100 in running buffer) were first flowed over reference (Fc1) surfaces to ensure low levels of non-specific binding at 25 μL/min. Next, diluted sera samples #1–5 (1/3000, 1/1000, 1/100) were injected over in-tandem reference and protein-immobilized flow cells at 25 μL/min (i.e., 5 min capture of nAb to Sp surfaces followed by ‘Wash: needle’ and ‘Wash: IFC’ commands) prior to the fixed ACE2 injections (100 nM × 5 min association +15 min dissociation) and regeneration.

### Quantification and statistical analysis

#### Quantification of viral RNA loads (hamster model)

Extraction of RNA from nasal washes was performed using the QIAamp Viral RNA Mini Kit Cat No./ID: 52906) according to the manufacturer’s instructions. Extraction of RNA from lung lobes and nasal turbinate was performed with approximately 100 μg of tissue, which was first homogenized in 600 μL of lysis buffer (RLT Qiagen) with a sterile stainless-steel bead in the TissueLyserII (Qiagen) and then RNA subsequently extracted using the Qiagen RNeasy Mini Kit (Cat No/ID: 74106)) according to the manufacturer’s instructions. The RT-qPCR assays were performed on RNA from samples of nasal washes, lung tissues, and nasal turbinate using SARS-CoV-2 specific primers targeting the env gene of the Wuhan strain. The forward Primer sequence is: ACAGGTACGTTAATAGTTAATAGCGT, whereas the Reverse Primer sequence is ATATTGCAGCAGTACGCACACA. The Probe used in this assay is: FAM-ACACTA-ZEN-GCCATCCTTACTGCGCTTCG-IBFQ (Integrated DNA technologies). Qiagen Quantifast RT-PCR Probe kits were used for RT-qPCR and samples were run on the OneStep Plus (Applied Biosystems) using the following program: reverse transcription (RT) 10 min at 50°C; inactivation 5 min at 95°C followed by 40 cycles of denaturation for 10 s at 95°C and annealing/extension for 30 s at 60°C. Results were expressed as copy number per reaction by generating a standard curve using with linearized plasmid DNA that contains the env gene of SARS-CoV-2 the Wuhan strain. The Ct values for individual samples were used with the standard curve to determine the copy number in each sample.

#### Infectious virus quantification (Hamster model)

Viral titration assays were performed to determine the level of infectious virus. The assays were conducted in 96-well plates using Vero’76 cells (ATCC CRL-1587). Median TCID_50_ was determined by microscopic observation of CPE of cells. The virus was quantified and reported in TCID_50_/mL or TCID_50_/gram. TCID_50_ values were calculated using the Spearman & Karber method in Excel.^60^^,^^61^

#### Cryo-EM image processing

All processing was performed in cryoSPARC unless otherwise noted.^62^ About 6,632 movies were collected, and motion corrected using patch motion correction. The contrast transfer functions (CTFs) of the flattened micrographs were determined using patch CTF and an initial stack of particles were picked using blob picker. Successive rounds of reference-free 2D classification were performed to generate clean particle stacks. These particles were used for ab-initio reconstruction to generate an initial model. This model was refined and subsequently the particles were used in alignment-free 3D classification to sort between the various states of receptor binding domain (RBD) conformation. A large majority of the particles displayed a conformation with 2 RBD “down” and 1 RBD “up”. These particles were then refined using non-uniform refinement^63^) and a local refinement with a generous mask around the entire complex. This resulted in a 3.1 Å reconstruction of Accum®-Spike which was sharpened using EMReady.^64^

#### Cryo-EM model building and refinement

A model of Spike protein, with a similar RBD arrangement to that which was observed, (PDB 7WK3) was docked into the map using UCSF Chimera X.^65^ This was followed by automated refinement using Phenix real space refine^66^ and Namdinator^67^ as manual building in Coot.^68^ The final model produced a favourable MolProbity score of 1.91 with 93.19% Ramachandran favored and 0.30% outliers.

#### SPR data analysis

The data obtained were doubled-referenced, and representative of duplicate injections acquired from at least three independent trials. ACE2-only injections at the start and end of each series were compared to ensure consistent SPR surface activity throughout the analyses. Apparent equilibrium dissociation constants (*K*_D_) were determined by global fitting of the titration data to the “1:1 kinetic” model in the BIAevaluation v4.1 software.

#### Statistical analysis

*p-*Values were calculated using one-way analysis of variance (ANOVA) or Log rank test for animal survival experiments. Results are represented as average mean with standard deviation (S.D.) error bars, and statistical significance is represented with asterisks: ∗*p* ˂ 0.05, ∗∗*p* ˂ 0.01, ∗∗∗*p* ˂ 0.001.
