# A Spike-Accum bioconjugate protein vaccine confers potent SARS-CoV-2-specific immunity

**DOI:** 10.1016/j.isci.2025.113457

**Published:** 2025-09-12

**Authors:** Jean Pierre Bikorimana, Nathanael A. Caveney, Nehme EL-Hachem, Gabrielle A. Mandl, John A. Capobianco, Daniela Stanga, Jamilah Abusarah, Mark A. Hancock, Roudy Farah, Marina P. Gonçalves, Darryl Falzarano, Mingmin Liao, Glenn Hamonic, Qiang Liu, Simon Beaudoin, Sebastien Talbot, Moutih Rafei

## Main text

(iScience *28*, 113314; September 19, 2025)

During copyediting, the production team mistakenly changed “A” to “An” in the title “A Spike-Accum bioconjugate protein vaccine confers potent SARS-CoV-2-specific immunity.” This has now been updated online. The production team apologizes for this error.

