# Supplementary material for: A Spike-Accum bioconjugate protein vaccine confers potent SARS-CoV-2-specific immunity
Source: iScience. 2025 Aug 7;28(9):113314. doi: 10.1016/j.isci.2025.113314 (PMC12496170; doi:10.1016/j.isci.2025.113314)
Supplement: Document S1. Figures S1–S12 and Table S1 [file mmc1.pdf]

iScience, Volume *28*

Supplemental information

A Spike-Accum bioconjugate protein vaccine confers potent SARS-CoV-2-specific immunity

Jean Pierre Bikorimana, Nathanael A. Caveney, Nehme EL-Hachem, Gabrielle A. Mandl, John A. Capobianco, Daniela Stanga, Jamilah Abusarah, Mark A. Hancock, Roudy Farah, Marina P. Gonçalves, Darryl Falzarano, Mingmin Liao, Glenn Hamonic, Qiang Liu, Simon Beaudoin, Sebastien Talbot, and Moutih Rafei

SUPPLEMENTARY FIGURES

A

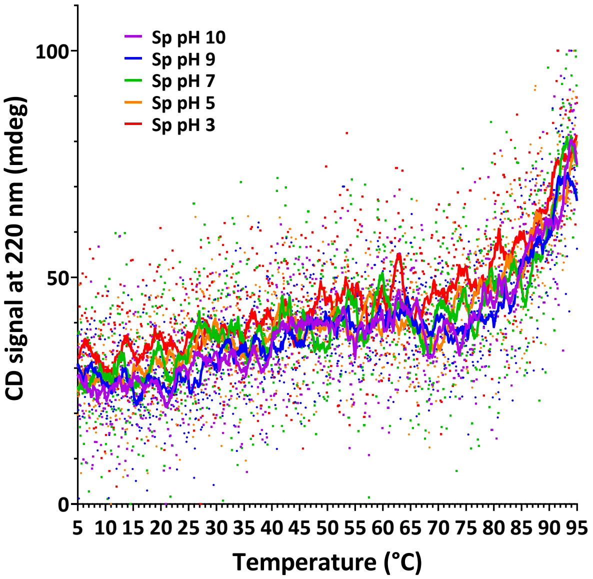

B

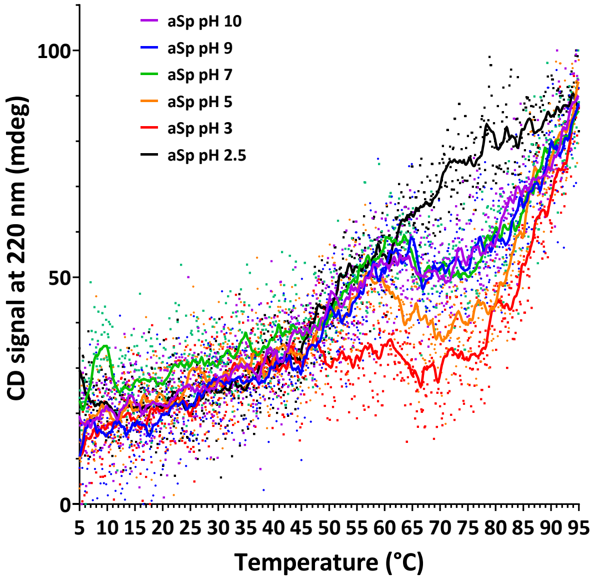

Figure S1. Biochemical characterization of unmodified Sp versus aSp. Related to Figure 1. Temperature-dependent stability of Sp (**A**) and aSp (**B**) as a function of pH as evidenced by changes in CD signal at 220 nm.

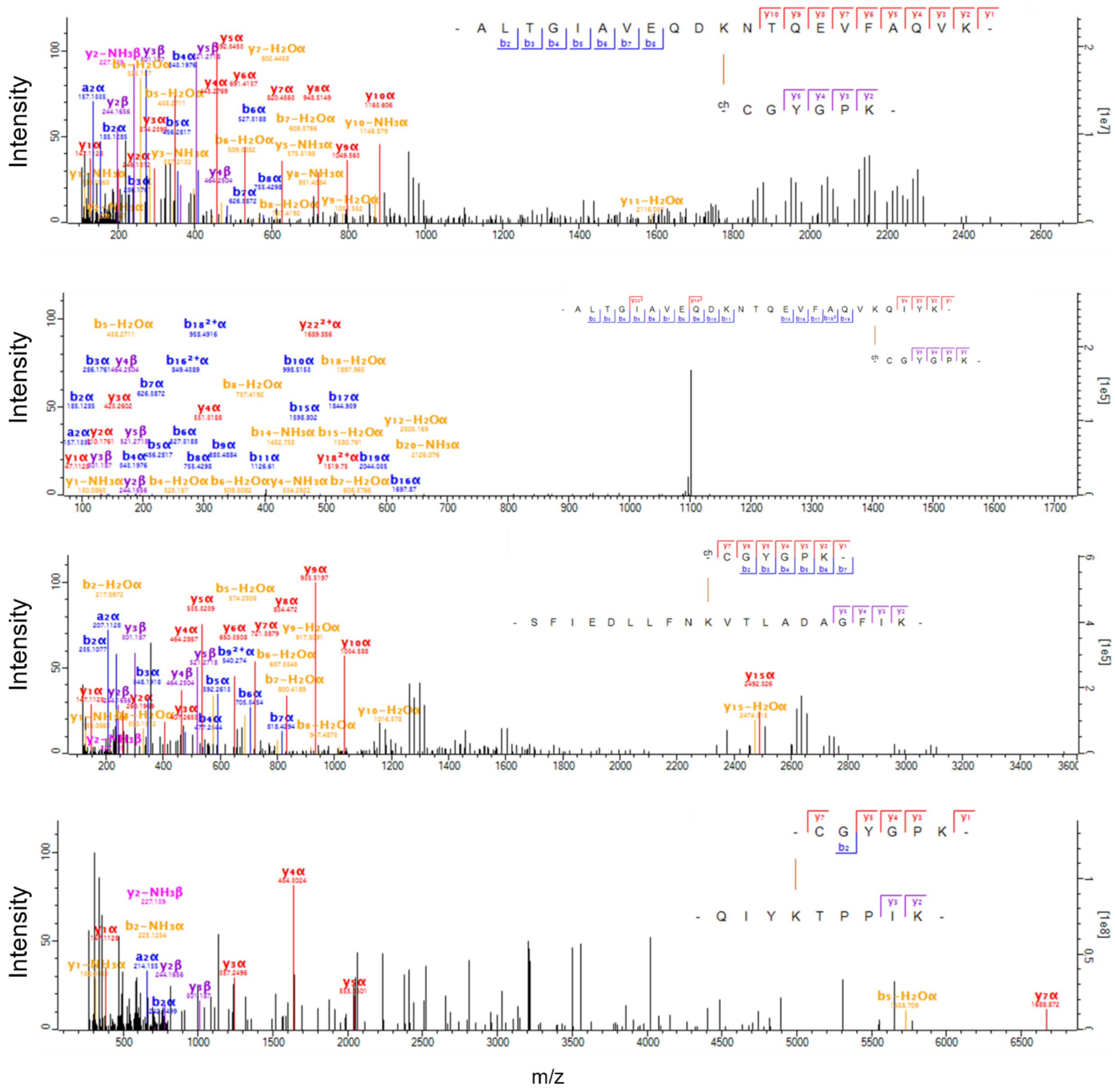

Figure S2. Mass spectra of the amino acid sequences found to be most commonly modified by Accum. Related to Figure 1. Each spectrum maps the Spike-derived peptide sequence that was modified by the Accum sequence (CGYGPK).

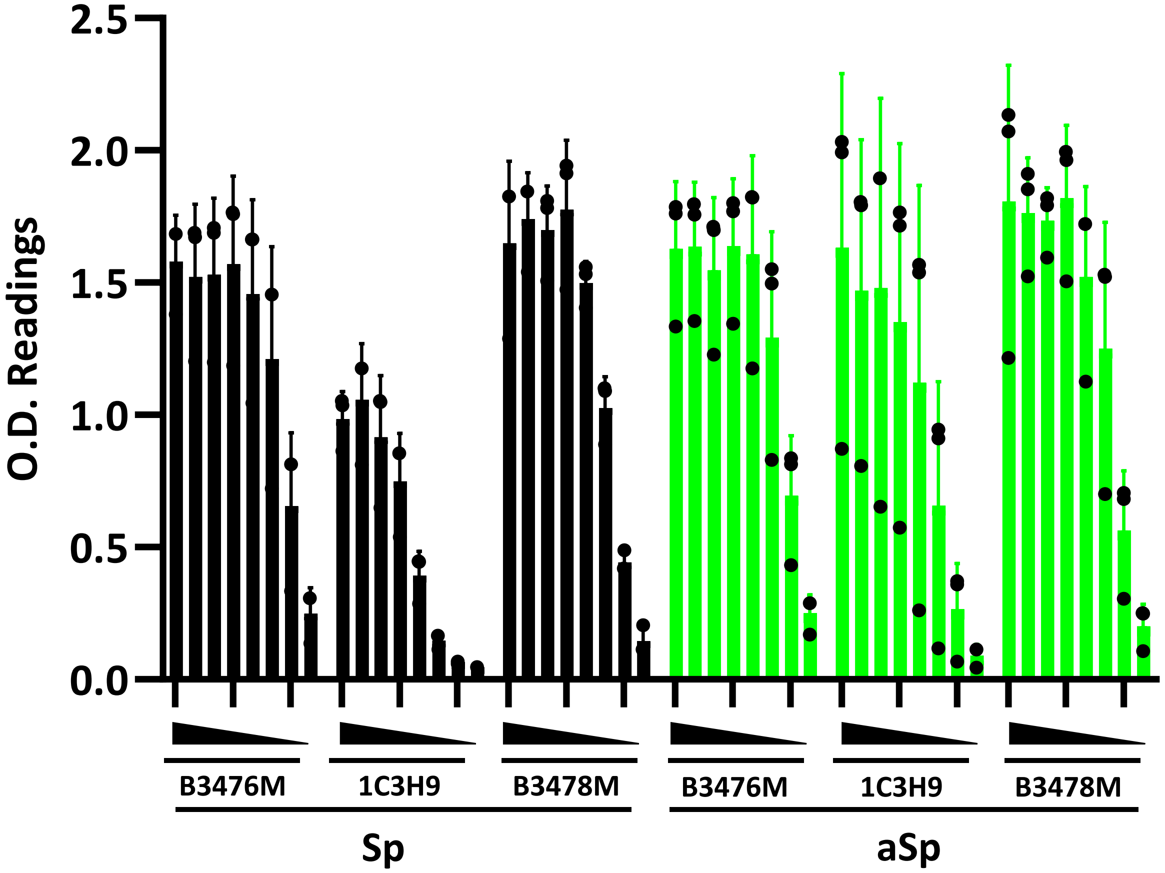

Figure S3. ELISA conducted on Sp versus aSp using three different commercial Sp-specific mAb. Related to Figure 1. ELISA readouts using the native Sp protein are shown in black whereas those conducted on aSp are shown in green. The dilutions used for the three mAb are: 2000, 1000, 500, 250, 50, 10, 2, and 1 ng/ml. Data are represented as mean +/- SD.

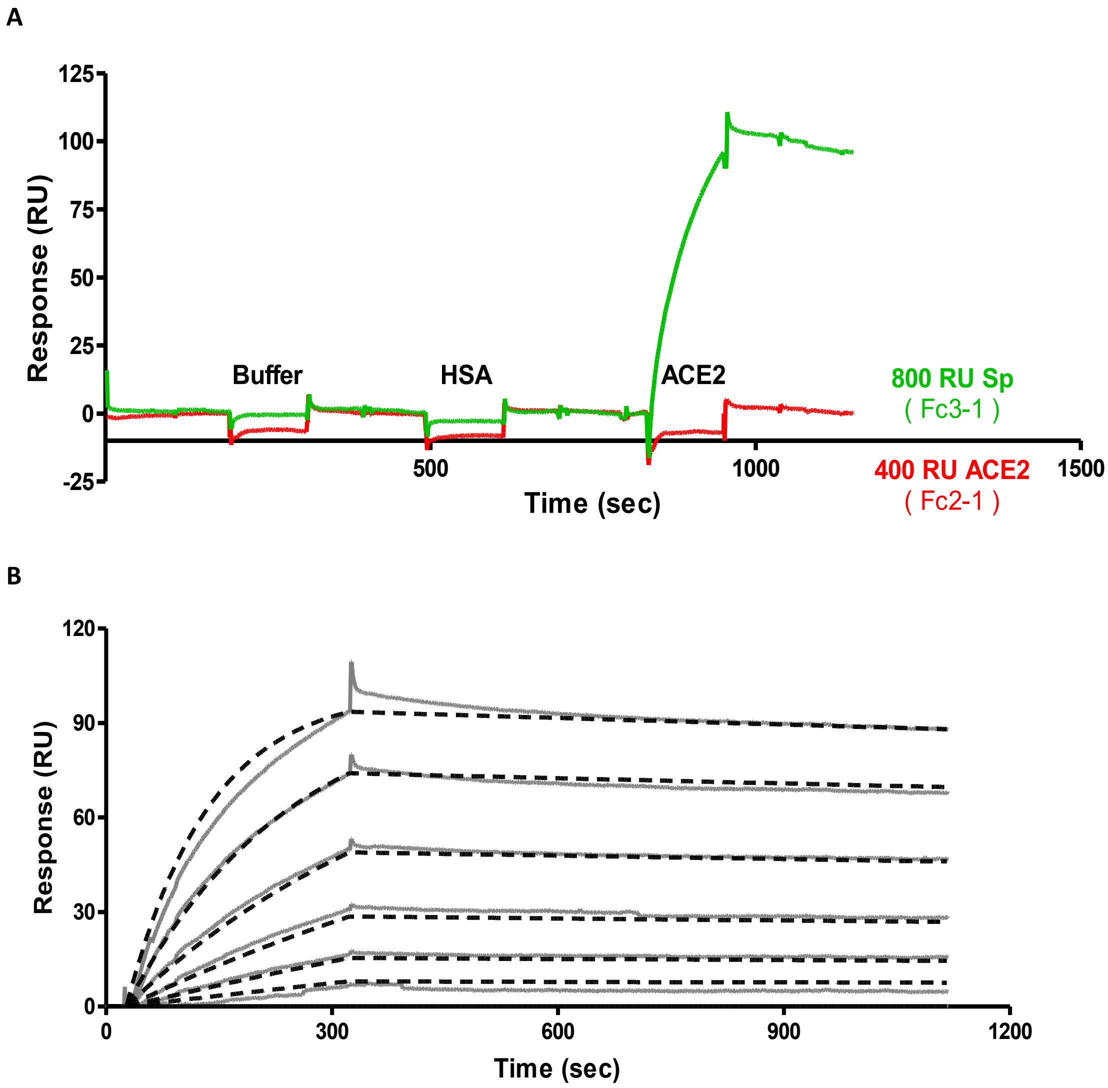

Figure S4. Specific, dose-dependent binding of ACE2 receptor to immobilized Sp, as assessed by SPR. Related to Figure 2. A) Representative specificity of successive 2 min injections of buffer (blank), 250 nM human serum albumin (negative), and 250 nM ACE2 flowing over low-density, amine-coupled ACE2 (lower red, Fc2-1) or Sp (upper green, Fc3-1) surfaces at 25 µL/min. **B)** Representative dose-dependent binding of ACE2 (0 – 250 nM; 2-fold dilution series) to amine-coupled Sp (800 RU) at 25 µL/min (5 min association + 15 min dissociation) predicts low nM affinity (K_D = 2 +/- 0.1 nM); solid grey lines represent raw data and black dashed lines represent global fit to “1:1 kinetic” model in BIAevaluation software.

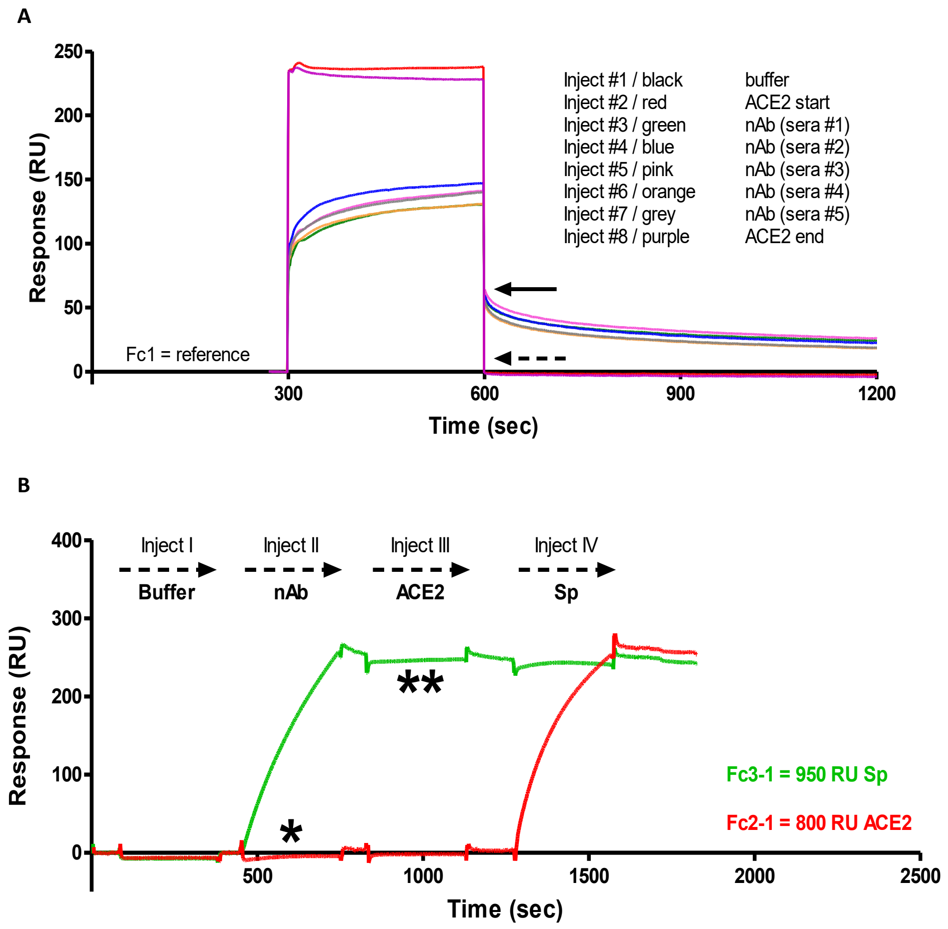

Figure S5. Binding of neutralizing antibody (nAb)-containing sera to immobilized Sp blocks ACE2 receptor interaction, as assessed by SPR. Related to Figure 2. A) Representative sequence of injected buffer (blank), ACE2 (100 nM), or sera #1-5 (diluted 1/100 each) over Fc1 (no protein reference surface) at 25 uL/min (5 min association + 10 min dissociation) to test for non-specific binding (dashed arrow <5 RU; black arrow <60 RU). **B)** Representative in-tandem injections of buffer (blank), nAb (1/200 diluted NP sera), ACE2 (100 nM), and Sp (100 nM) flowing over amine-coupled ACE2 (*lower red*, Fc2-1) or Sp (*upper green*, Fc3-1) surfaces at 25 uL/min. Note 1: *nAb does not bind to coupled ACE2 surface unlike downstream Sp positive control. Note 2: **nAb bound to coupled Sp surface blocks subsequent ACE2 binding.

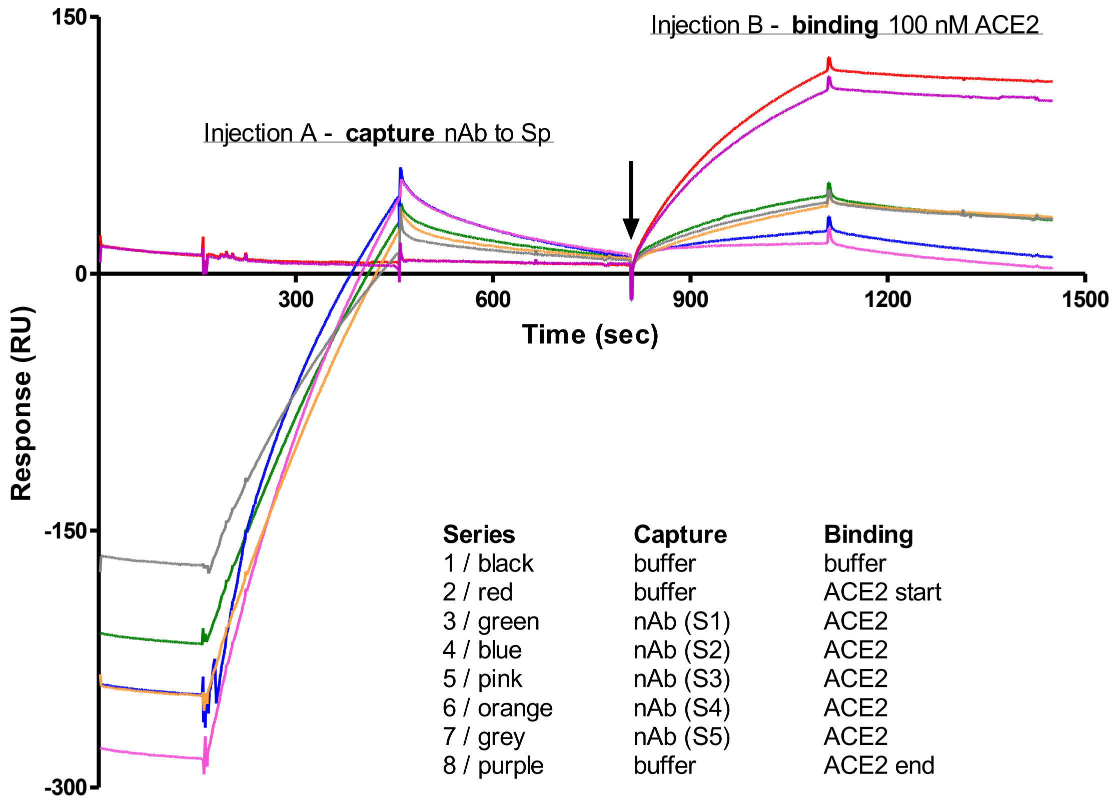

Figure S6. Development of optimized SPR assay to block ACE2 receptor binding to Sp in the presence of neutralizing antibody (nAb)-containing sera. Related to Figure 2. Representative sensorgrams for in-tandem "Capture" (inject 1/200 diluted sera #1-5) and "Binding" (inject buffer or 100 nM ACE2) at 25 uL/min (5 min capture + 5 min binding) to test for sera that attenuate ACE2 binding to amine-coupled Sp (950 RU). For clarity, sensorgrams were re-aligned at 800 sec (black arrow) to compare head-to-head capture (170-280 RU bound nAb) and binding (maximal ACE2 responses at start or end in the absence of bound nAb).

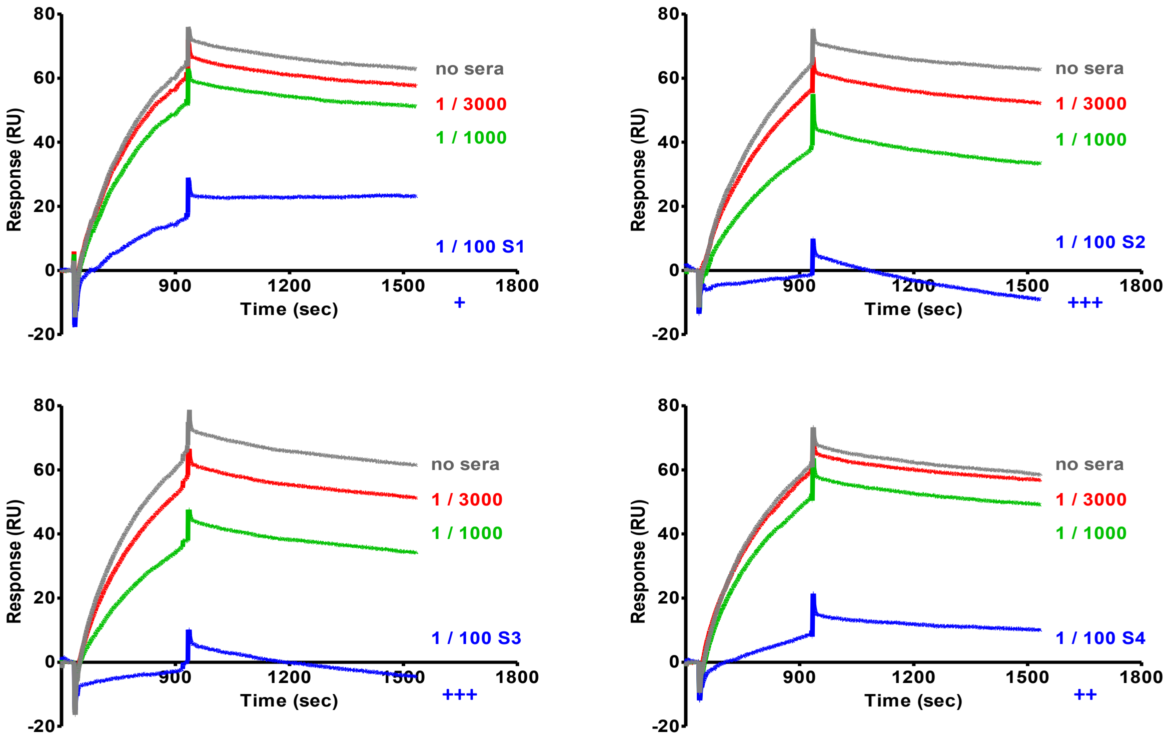

Figure S7: Neutralizing antibody (nAb) sera exhibit dose-dependent inhibition of binding between ACE2 receptor and immobilized Sp (950 RU) in optimized SPR assay. Related to Figure 2. Representative sensorgrams for sequential "Capture" (buffer or diluted sera; <600 sec) and "Binding" (inject buffer or 100 nM ACE2; >600 sec) at 25 uL/min (5 min association + 10 min dissociation). Panels: representative screening of neutralizing antibody sera #2-5 (fixed dilution series) to detect relative inhibition (+ strong; ++ stronger; +++ strongest) of ACE2-Sp binding interaction; inhibitory effect of neutralizing antibodies (at 1/100 or 1/1000 dilutions) was not observed at 1/3000 dilutions.

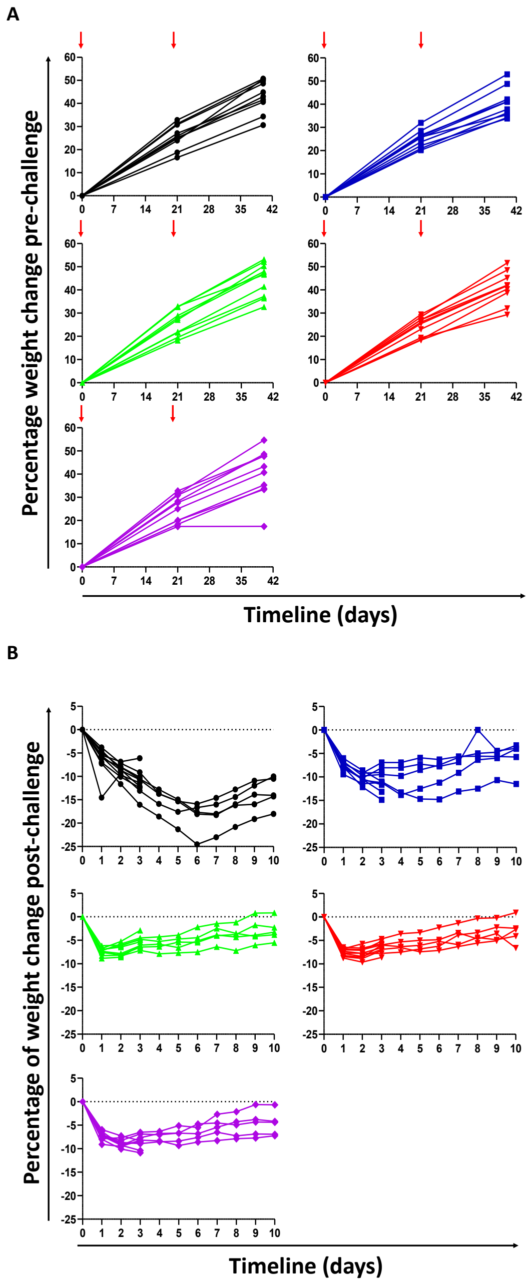

Figure S8. Monitoring of hamster body weights pre- and post-challenge. Related to Figure 6. A) Body weight gain of hamsters during the immunization phase. Each line represents one animal: control (in black); dose 1 (in blue); dose 2 (in green): dose 3 (in red): dose 2 (P) (in pink). **B)** Percent body weight loss of hamsters after SARS-CoV-2 challenge. The same color coding used in panel (A) applies to panel B.

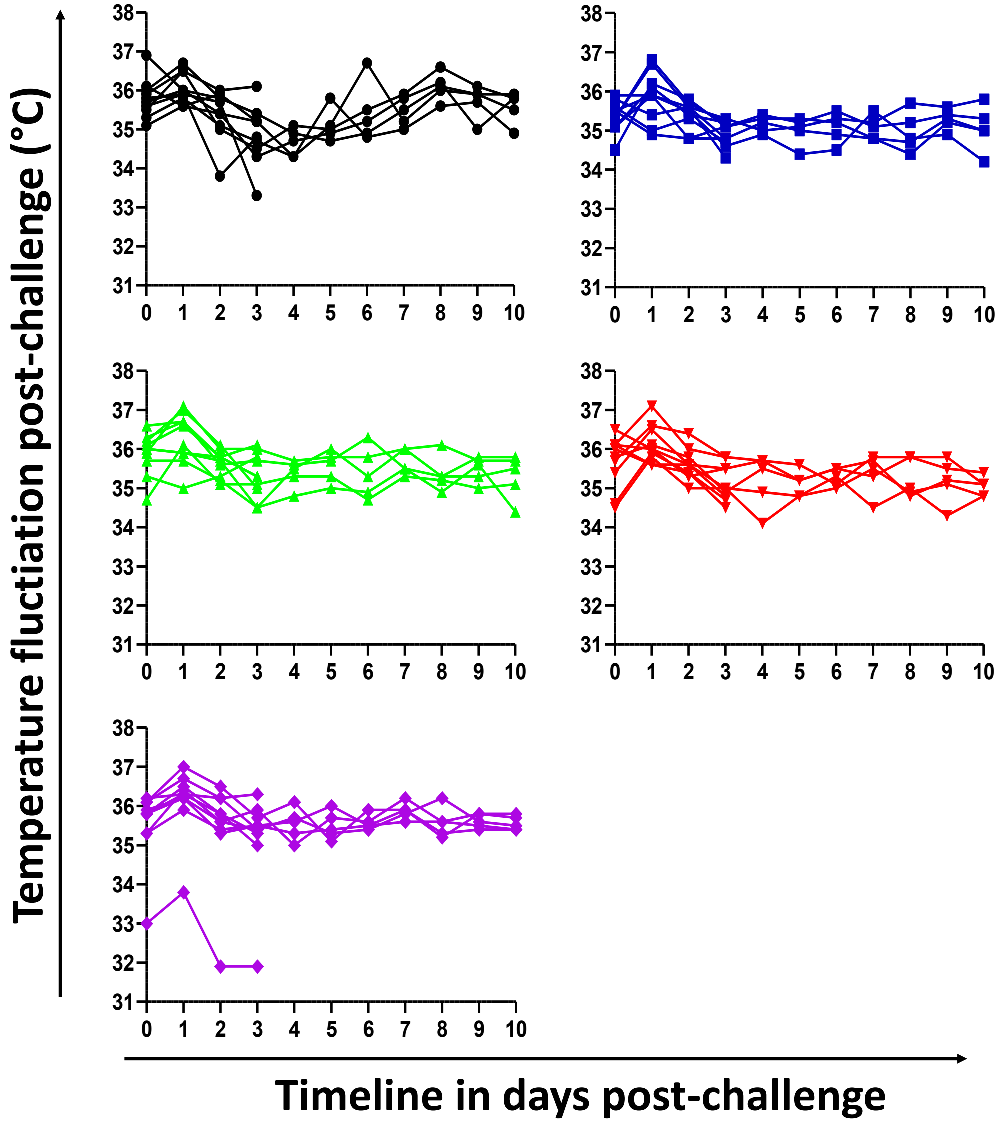

Figure S9. Subcutaneous temperature fluctuations in hamsters after SARS-CoV-2 challenge. Related to Figure 6. Fluctuations in temperature post-challenge was conducted on a daily basis until the end of the experiment. Control (in black); dose 1 (in blue); dose 2 (in green); dose 3 (in red); dose 2 (P) (in pink).

A

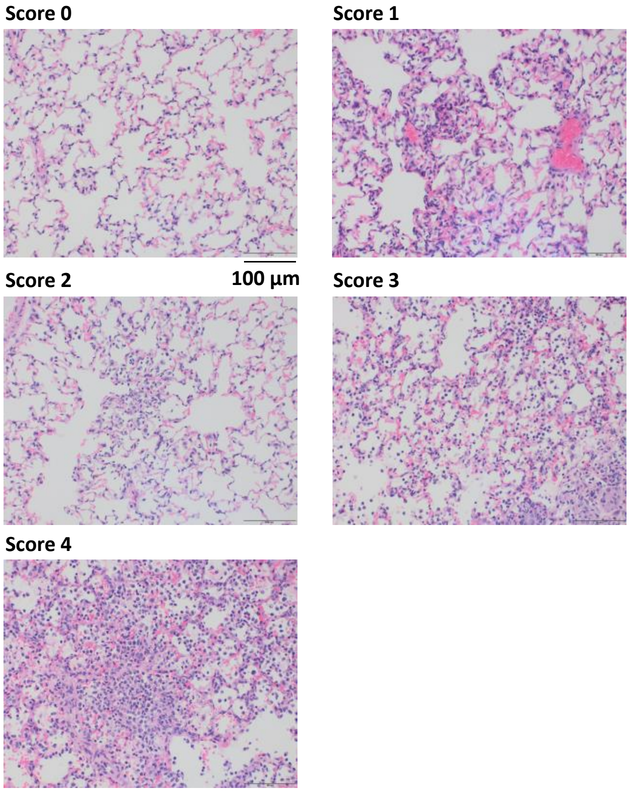
Score 0
Score 1
100 µm
Score 2
Score 3
Score 4

B

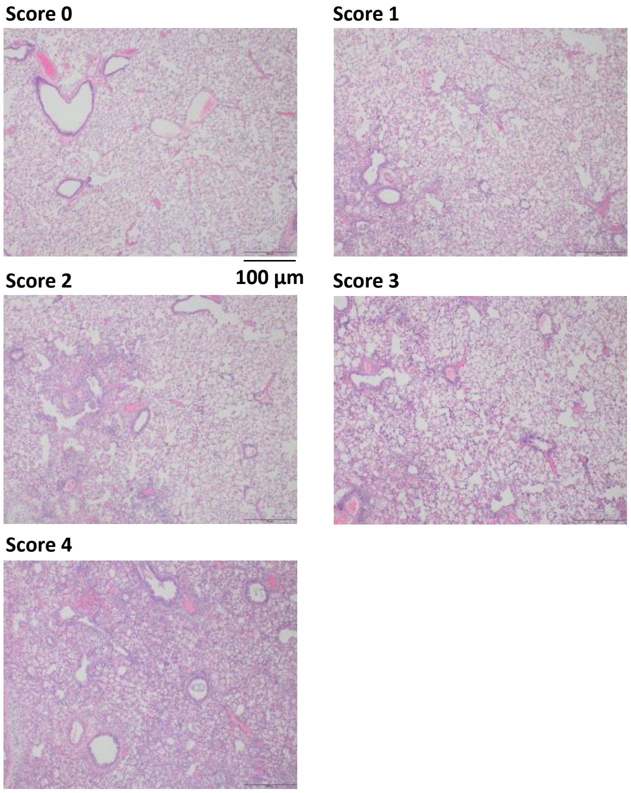
Score 0
Score 1
100 µm
Score 2
Score 3
Score 4

Figure S10. Lung and body weights after challenge at 3 and 10 dpc. Related to Figure 6. A) Fluctuations in lung weight (top panel) versus fluctuation per total body weight. The analysis was conducted at 3 dpc. Each dot represents one animal. Control (in black); dose 1 (in blue); dose 2 (in green): dose 3 (in red): dose 2 (P) (in pink). B) Same as (A) except that it was conducted at 10 dpc. Scale bar represents 100 μm.

A

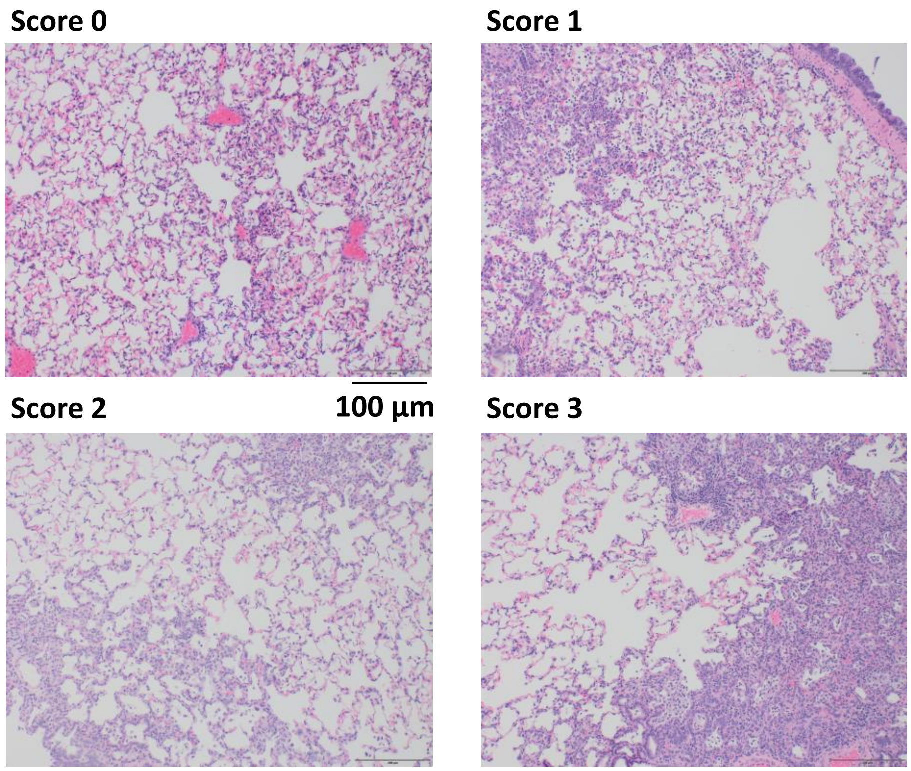
Score 0
Score 1
Score 2
100 µm
Score 3

B

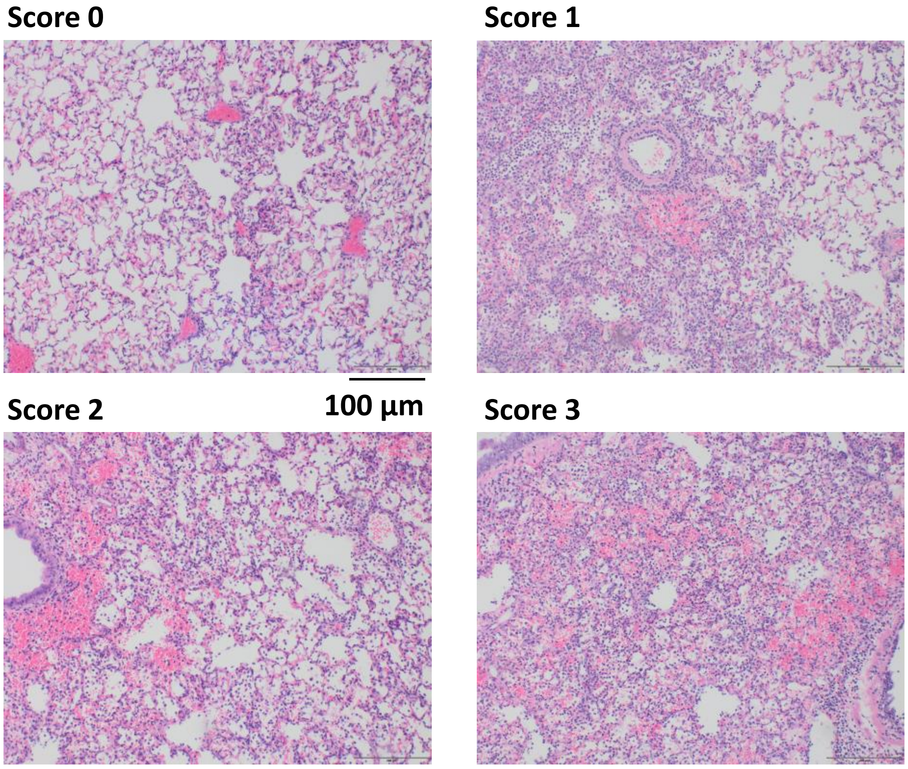
Score 0
Score 1
Score 2
100 µm
Score 3

Figure S11. Histological analyses for severity of inflammation in hamster lungs. Related to Figure 6. A) Representative staining for inflammation in the lungs. Score 0: absent inflammation; Score 1: inflammation minimal; Score 2: inflammation mild; Score 3: inflammation moderate; Score 4: inflammation marked. **B)** Representative staining of affected parenchyma. Score 0: absent 0%; Score 1: 1-24%; Score 2: 25-49%; Score 3: 50-74%; Score 4: 75-100%. Scale bar represents 100 μm.

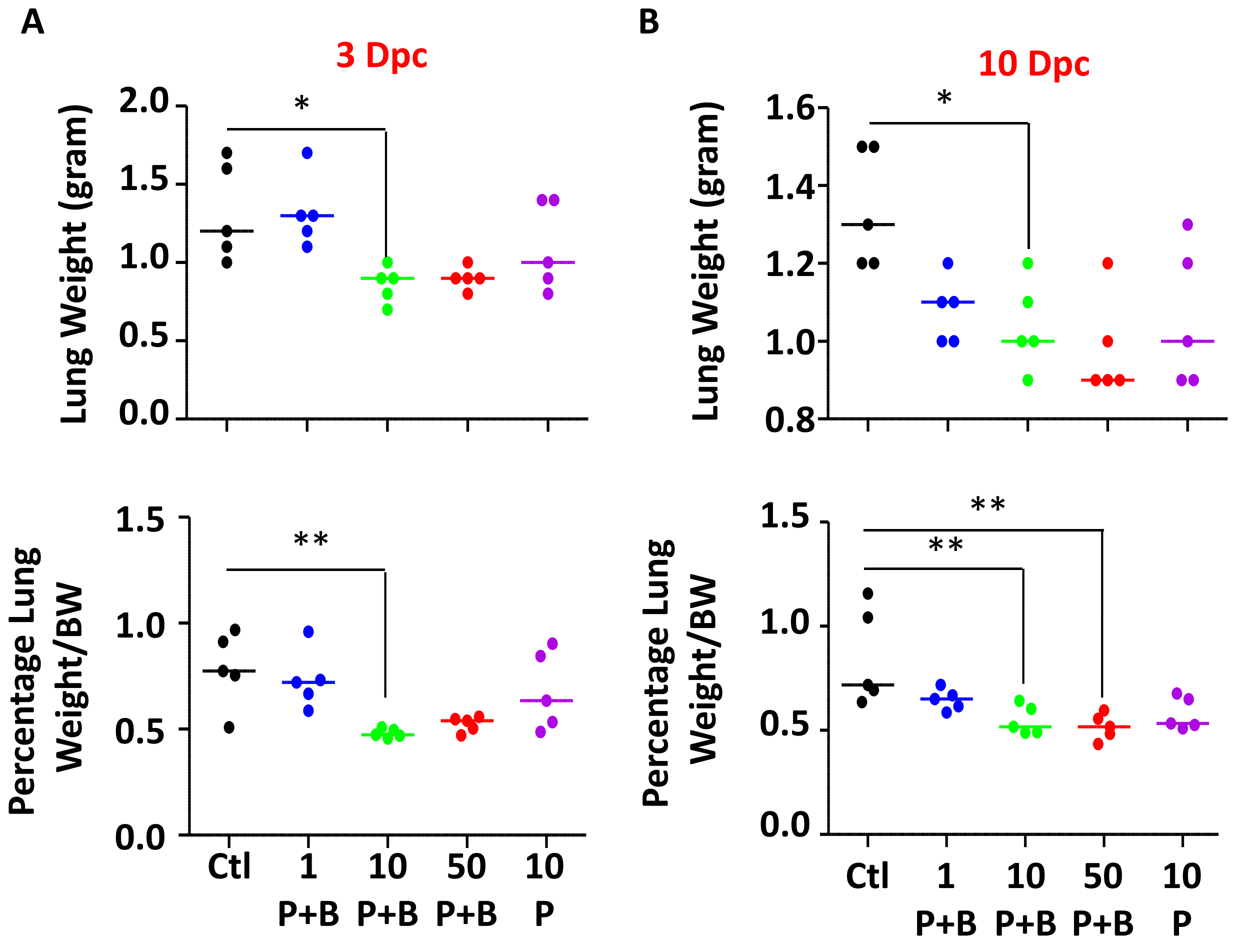

Figure S12. Histological analyses of pneumocyte hyperplasia and hemorrhage extent in hamster lungs. Related to Figure 6. A) Representative staining for pneumocyte hyperplasia in the lungs. Score 0: absent 0%; Score 1: 1-24%; Score 2: 25-49%; Score 3: 50-74%; Score 4: 75-100%. **B)** Representative staining for severity of hemorrhage in the lungs. Score 0: absent 0%; Score 1: 1-24%; Score 2: 25-49%; Score 3: 50-74%; Score 4: 75-100%. Data are represented as mean +/- SD with **p<0.01 and ***p<0.001.

Sequence	Length	Mass	Intensity (Sp)	Intensity (aSp)	Conjugation Level
ALTGIAVEQDKNTQEVFAQVK	21	2288.2012	6.09E+10	1.48E+10	0.20
ALTGIAVEQDK	11	1143.6136	1.93E+09	2.79E+08	0.34
QIYKTPPIK	9	1086.6437	1.24E+08	1.30E+07	0.10
SFIEDLLFNKVTLADAGFIK	20	2240.2093	1.50E+09	1.82E+08	0.11
SFIEDLLFNK	10	1224.639	2.20E+10	1.21E+10	0.35
				**DAR_{avg}**	3.3

SUPPLEMENTARY TABLE

Table S1: Liquid chromatography tandem mass spectrometry (LC MS/MS) data corresponding to the modified sequences. Related to Figure 1. The table shows the intensity for each identified sequence obtained for unconjugated Spike (Sp) and Accum conjugated Spike (aSp) samples with the corresponding DAR contribution.
